# Climate Stability Index maps, a global high resolution cartography of climate stability from Pliocene to 2100

**DOI:** 10.1038/s41597-022-01144-5

**Published:** 2022-02-10

**Authors:** Sonia Herrando-Moraira, Neus Nualart, Mercè Galbany-Casals, Núria Garcia-Jacas, Haruka Ohashi, Tetsuya Matsui, Alfonso Susanna, Cindy Q. Tang, Jordi López-Pujol

**Affiliations:** 1 Botanic Institute of Barcelona (IBB, CSIC-Ajuntament de Barcelona), Pg. del Migdia, s.n., 08038 Barcelona, Spain; 2 Systematics and Evolution of Vascular Plants (UAB) – Associated Unit to CSIC, Departament de Biologia Animal, Biologia Vegetal i Ecologia, Facultat de Biociències, Universitat Autònoma de Barcelona, 08193 Bellaterra, Spain; 3 Forestry and Forest Products Research Institute, Forest Research and Management Organization, Matsunosato 1, Tsukuba-shi, Ibaraki-ken, 305-8687 Japan; 4 Faculty of Life and Environmental Sciences, University of Tsukuba, Tennodai 1-1-1, Tsukuba, Ibaraki, 305-8572 Japan; 5 Institute of Ecology and Geobotany, College of Ecology and Environmental Science, Yunnan University, Dongwaihuan South Road, University Town, Chenggong New District, Kunming, Yunnan 650504 China

**Keywords:** Projection and prediction, Biogeography

## Abstract

Climate changes are top biodiversity shapers, both during the past and future. Mapping the most climatic stable and unstable zones on Earth could improve our understanding of biodiversity distribution and evolution. Here, we present a set of maps based on a global scale, high resolution (ca. 5 km) new Climate Stability Index (CSI). The CSI considers bioclimatic variables for two different time ranges: (1) from Pliocene (3.3 Ma) to the present (CSI-past map set), using 12 time periods of *PaleoClim* representing warm and cold cycles; and (2) from present to the year 2100 (CSI-future), using nine general circulation models of climate change of four periods available from *WorldClim*. We calculated standard deviation of the variables and selected an uncorrelated set for summing, normalizing and obtaining the CSI maps. Our approach is useful for fields such as biogeography, earth sciences, agriculture, or sociology. However, CSI is an index that can be re-calculated according to particular criteria and objectives (e.g. temperature variables); maps are, therefore, customizable to every user.

## Abstract (summary)

**Table:** 

Measurement(s)	climate stability index
Technology Type(s)	general circulation models of climate change
Factor Type(s)	temporal interval
Sample Characteristic - Environment	climate system • climate change
Sample Characteristic - Location	global

Machine-accessible metadata file describing the reported data: 10.6084/m9.figshare.19050569

## Background & Summary

Long-term climatic variation has had an enormous impact on the evolution of biodiversity, including humans. Climatic instability linked to the Pleistocene glacial/interglacial cycles has caused active diversification^1–3^. In contrast, regions with relatively stable climates have often acted either as “museums” (places of persistence)^4,5^ or both as “museums” and “cradles”^6,7^. In addition, recent global warming is causing even deeper changes in biodiversity in a very short space of time. Increases of temperature in just a few decades might produce large regional species turnover by increasing extinction rates and large migrations^8^, and also by loss of phylogenetic diversity (= evolutionary potential)^9^.

Identifying areas with high climatic stability is of enormous interest. Delimiting past trends of stability and mapping them can help to shed light into evolutionary processes that shaped current biota, including human evolution. They may also be helpful in designing new protected areas (PAs) and prioritizing or redesigning the existing ones^10^, following the demand of incorporating evolutionary processes into conservation planning^11^. Mapping climatic stability for the future can be even more important, as climate patterns are predicted to change dramatically during this century, including the occurrence of extreme weather events^8,12^. Such mapping may have, thus, implications on economy and human health; e.g., when selecting new arable lands or new human settlements that will be needed as a consequence of the predicted large-scale migrations^8^.

Attempts to cartography climate stability are not new. The only ones that offer data at a global scale are *StableClim*^13^ and the “map of climate stability”^14^ (hereinafter “MCS”), which are projections of climate variation, the former from 21,000 BP (LGM or Last Glacial Maximum) to 2100 CE^13^, and the latter from 21,000 BP to 100 BP^14^. *StableClim* offers estimates of climate stability on an ideal time-scale (every 100 years) for surveying the eco-evolutionary impacts of short-term climate shifts^13^, but it shares with MCS three limitations^14^: (1) coverage of only ca. 21,000 years, which hinders their utility for explaining the role of climate stability in evolution and speciation that take thousands or millions of years; (2) spatial resolution is insufficient (2.5° grid, ca. 278 km at the equator), which does not allow one to correlate climatic stability with population data on both local and regional scales; and (3) they use one or two variables (mean monthly temperatures^13^ or mean monthly temperatures and precipitations^14^) that may not be the key ones for a given case of study; for example, distribution patterns of bees in South Africa are highly dependent on seasonality and not on mean rainfall values^15^.

**Fig. 1:**
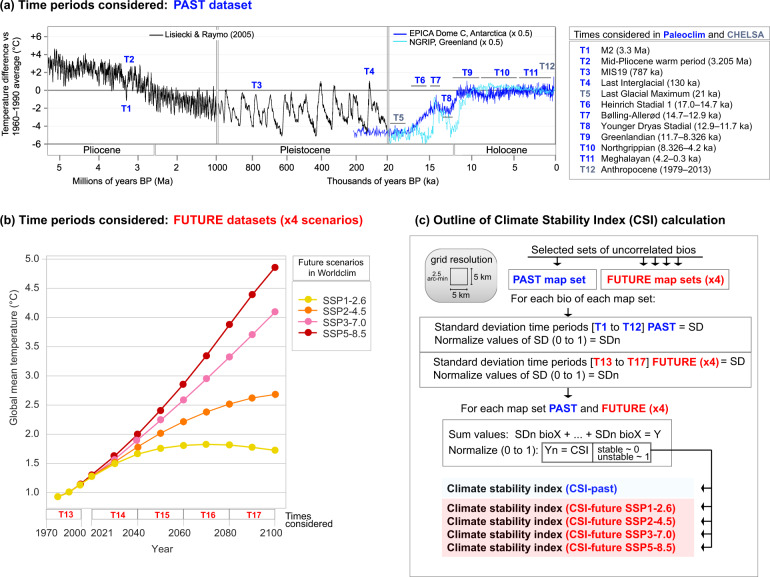
General overview of time periods considered to calculate the Climate Stability Index (CSI) for past and future map sets and graphical illustration of index generation. (**a**) Time periods used to estimate the CSI-past and from which paleoclimate data have been taken (from *PaleoClim*^16^ database, which includes two map sets from CHELSA^25^), represented by a Pliocene to Holocene temperature reconstruction modified from https://en.wikipedia.org/wiki/Geologic_temperature_record. Temperature reconstruction showed in a black line derived from Lisiecki and Raymo^26^. (**b**) Time periods and four Shared Socioeconomic Pathways (SSPs: SSP1-2.6, SSP2-4.5, SSP3-7.0, and SSP5-8.5) considered to estimate CSI-future using climate data from *WorldClim*^17^, modified from Gidden *et al*.^27^. (**c**) Schematic representation of the methodology employed to calculate the CSI for each map set.

Thanks to the recent publication of *PaleoClim*^16^, a free-access database of paleoclimate layers at 2.5 arc-min (~5 km) grid resolution that includes data much older than the LGM, we are presenting a new set of maps that are based on a new Climatic Stability Index (CSI). Given the markedly different speed of inferred climatic changes (see above), we divide the sets of maps into two: CSI-past and CSI-future. CSI-past is based on the 12 time periods (listed in Fig. 1a) included in *PaleoClim*^16^, which span from 3.3 Ma (Pliocene) to present. CSI-future uses the average values of nine general circulation models (GCMs) along four time intervals until 2100 (Fig. 1b), available in *WorldClim*^17^. CSI-future offers maps of climate stability for four Shared Socioeconomic Pathways (SSP^18^; Fig. 1b) that will be used to produce the IPCC Sixth Assessment Report on climate change. Variables for CSI calculation (Table 1) include annual averages (e.g. annual mean temperature), extremes (e.g. minimum temperature of coldest month), and seasonality (e.g. annual range in temperatures). CSI-past takes into account 14 bioclimatic variables, while CSI-future uses five more.

**Table 1:** Bioclimatic variables used to generate the Climate Stability Index (CSI) maps.

Variable	Unit	Map set (source)	Included in CSI maps
**Bio1**: Annual mean temperature	°C	Past (PaleoClim)	Past
		Future (WorldClim v2)	Future (SSP3)
**Bio2**: Mean diurnal range (Mean of monthly (max. temp. - min. temp.))	°C	Future (WorldClim v2)	Future (SSP1, SSP2, SSP3, SSP5)
**Bio3**: Isothermality (bio2/bio7) (×100)	dls	Future (WorldClim v2)	Future (SSP1, SSP2, SSP3, SSP5)
**Bio4**: Temperature seasonality (standard deviation ×100)	°C	Past (PaleoClim)	Past
		Future (WorldClim v2)	
**Bio5**: Max. temperature of warmest month	°C	Future (WorldClim v2)	Future (SSP3)
**Bio6**: Min. temperature of coldest month	°C	Future (WorldClim v2)	Future (SSP1, SSP2)
**Bio7**: Temperature annual range (bio5-bio6)	°C	Future (WorldClim v2)	Future (SSP1, SSP5)
**Bio8**: Mean temperature of wettest quarter	°C	Past (PaleoClim)	Future (SSP1, SSP2, SSP3, SSP5)
		Future (WorldClim v2)	
**Bio9**: Mean temperature of driest quarter	°C	Past (PaleoClim)	Past
		Future (WorldClim v2)	Future (SSP1, SSP2, SSP3, SSP5)
**Bio10**: Mean temperature of warmest quarter	°C	Past (PaleoClim)	Future (SSP1, SSP2, SSP5)
		Future (WorldClim v2)	
**Bio11**: Mean temperature of coldest quarter	°C	Past (PaleoClim)	Past
		Future (WorldClim v2)	
**Bio12**: Annual precipitation	mm	Past (PaleoClim)	Past
		Future (WorldClim v2)	Future (SSP1, SSP3, SSP5)
**Bio13**: Precipitation of wettest month	mm	Past (PaleoClim)	Past
		Future (WorldClim v2)	Future (SSP3, SSP5)
**Bio14**: Precipitation of driest month	mm	Past (PaleoClim)	Past
		Future (WorldClim v2)	Future (SSP1)
**Bio15**: Precipitation seasonality (coefficient of variation)	dls	Past (PaleoClim)	Past
		Future (WorldClim v2)	Future (SSP1, SSP2, SSP3, SSP5)
**Bio16**: Precipitation of wettest quarter	mm	Past (PaleoClim)	Future (SSP2)
		Future (WorldClim v2)	
**Bio17**: Precipitation of driest quarter	mm	Past (PaleoClim)	Future (SSP2, SSP3, SSP5)
		Future (WorldClim v2)	
**Bio18**: Precipitation of warmest quarter	mm	Past (PaleoClim)	Past
		Future (WorldClim v2)	Future (SSP1, SSP2, SSP3, SSP5)
**Bio19**: Precipitation of coldest quarter	mm	Past (PaleoClim)	Past
		Future (WorldClim v2)	Future (SSP1, SSP2, SSP3, SSP5)

For each variable we include the following information: the units, whether they are included in a climate map set (and source from where it can be downloaded), and whether they have been taken into account to calculate the CSI. Note that bio2, bio3, bio5, bio6 and bio7 were not included for the calculation of CSI-past as these variables are not available in *PaleoClim* database^16^ for T1 (M2, Pliocene, ca. 3.3 Ma), T2 (mid-Pliocene warm period, Pliocene, 3.205 Ma), and T3 (MIS19, Pleistocene, ca. 787 ka). Unit abbreviations: °C (Celsius), mm (millimetres), dls (dimensionless).

In spite of the limitation of using only 12 time periods as a basis for inferring the long-term climatic stability (with only three older to 0.12 Ma), the much wider time interval, the variety of measures (means, peaks, and intra-year variability) and the finer resolution (ca. 5 km) makes our proposal a very versatile tool with a wide array of applications. With both CSI-past and CSI-future we offer maps of climatic stability adhering to FAIR principles^19^, adaptable to every user and circumstance: they are very easy to use and the variables to estimate the climate stability can be selected at the user’s discretion.

## Methods

A workflow for the calculation of CSI is presented in Fig. 1c. For all the analyses, we used the R v. 4.0.3 software environment^20^ implemented in RStudio v. 1.4.1103. The scripts used for each methodological step are available at the Figshare repository^21^. After data download from primary sources (*PaleoClim* and *WorldClim*), specifically for the CSI-future map set we performed an initial step aimed to obtain individual bioclimatic variables for each future time period for the four SSPs (Fig. 1b). To achieve this, the median values of nine GCMs were calculated in functions compiled in raster R package^22^ for each individual bioclimatic variable (see a few exceptions of number of GCMs used in Table 2).

**Table 2:** General circulation models (GCM) used to construct the future map sets.

2021–2040				
GCM	SSP1-2.6	SSP2-4.5	SSP3-7.0	SSP5-8.5
BCC-CSM2-MR	✓	✓	✓	✓
CNRM-CM6-1	✓	✓	✓	✓
CNRM-ESM2-1	✓	✓	✓	✓
CanESM5	✓	✓	✓	✓
GFDL-ESM4	✓	NA	✓	NA
IPSL-CM6A-LR	✓	NA	✓	✓
MIROC-ES2L	✓	✓	✓	✓
MIROC6	✓	✓	✓	✓
MRI-ESM2-0	✓	✓	✓	NA
**2041–2060**				
**GCM**	**SSP1-2.6**	**SSP2-4.5**	**SSP3-7.0**	**SSP5-8.5**
BCC-CSM2-MR	✓	✓	✓	✓
CNRM-CM6-1	✓	✓	✓	✓
CNRM-ESM2-1	✓	✓	✓	✓
CanESM5	✓	✓	✓	✓
GFDL-ESM4	✓	NA	✓	NA
IPSL-CM6A-LR	✓	✓	✓	✓
MIROC-ES2L	✓	✓	✓	✓
MIROC6	✓	✓	✓	✓
MRI-ESM2-0	✓	✓	✓	✓
**2061–2080**				
**GCM**	**SSP1-2.6**	**SSP2-4.5**	**SSP3-7.0**	**SSP5-8.5**
BCC-CSM2-MR	✓	✓	✓	✓
CNRM-CM6-1	✓	✓	✓	✓
CNRM-ESM2-1	✓	✓	✓	✓
CanESM5	✓	✓	✓	✓
GFDL-ESM4	✓	NA	✓	NA
IPSL-CM6A-LR	✓	✓	✓	✓
MIROC-ES2L	✓	✓	✓	✓
MIROC6	✓	✓	✓	✓
MRI-ESM2-0	✓	✓	✓	✓
**2081–2100**				
**GCM**	**SSP1-2.6**	**SSP2-4.5**	**SSP3-7.0**	**SSP5-8.5**
BCC-CSM2-MR	✓	✓	✓	✓
CNRM-CM6-1	✓	✓	✓	✓
CNRM-ESM2-1	✓	✓	✓	✓
CanESM5	✓	✓	✓	✓
GFDL-ESM4	✓	NA	✓	NA
IPSL-CM6A-LR	✓	✓	✓	✓
MIROC-ES2L	✓	✓	✓	✓
MIROC6	✓	✓	✓	✓
MRI-ESM2-0	✓	✓	✓	✓

Those GCM marked with “NA” correspond to non-available models in *WorldClim*^17^ database, and thus not used for the calculation of means and medians.

The standard deviation (SD) was estimated as a measure of the amount of variation or dispersion along time series, from which the resulting output maps showed the places where climate conditions remained constant or variable across the temporal periods considered (Fig. 1a,b). The SD, as a way to identify stable/unstable climatic areas, was previously used in other climatic or evolutionary studies^4,14^. To compute the SD output rasters, we applied the mosaic function setting “fun = sd” from raster R package, calculating the SD for each pixel in the 12 time period rasters for CSI-past and five times for CSI-future, independently for each variable. The mosaic function was also used for the range calculation, with “fun = min” and “fun = max” to obtain the minimum and maximum values of input rasters, respectively, with a further step for subtracting maximum to minimum values.

Specifically, for CSI-past, as it includes several time periods with sea-level dropping below the present level (T1, T3, T5, T6, T7, T8, T9; Fig. 1a), we applied a mask of the current land surface, i.e. taking the T12 (Anthropocene) as a template. With this additional step, we were able to remove those pixels (grid cells) currently under the sea but that were once emerged. Most of these pixels, however, were only emerged during the LGM (ca. 21 ka), thus having values for bioclimatic variables for just a single time period (instead of the 12 routinely used for the variability estimation). The inclusion of these areas would result in highly climatically stable regions (low SD values; Supplementary Fig. 1), but this would be an obviously biased result. In contrast, we did not remove those areas affected by the sea-level rising periods, as only three periods contained “NoData” values (T2, T4, T10; Fig. 1a). However, to take this fact into consideration, we created a raster file in which these areas submerged during warm periods are indicated (see Supplementary Fig. 1). Finally, for both CSI-past and CSI-future, the resulting SD values were normalized to values between 0 and 1, with 0 representing completely stable areas and 1 the most unstable ones.

The next step was focused on the selection of a relatively uncorrelated set of variables for each map set. We used the removeCollinearity function from virtualspecies R package^23^ that estimates the correlation value among pairs of variables from a given number of random sample points (10,000 in present case) according to a given method (Pearson for the present case) and a threshold of statistic selected (*r* > 0.8 as a cut-off value). The function removeCollinearity returns a list of uncorrelated variables according to the settings specified, randomly selecting just one variable from groups of correlated ones (see Table 1 for a complete list of variables used for each map set). As we compiled estimates of variability independently for each variable and map set (e.g. SD bio1 past, SD bio2 past, etc.), each user can define his own CSI, selecting the more interesting variables according to the case of study.

**Fig. 2:**
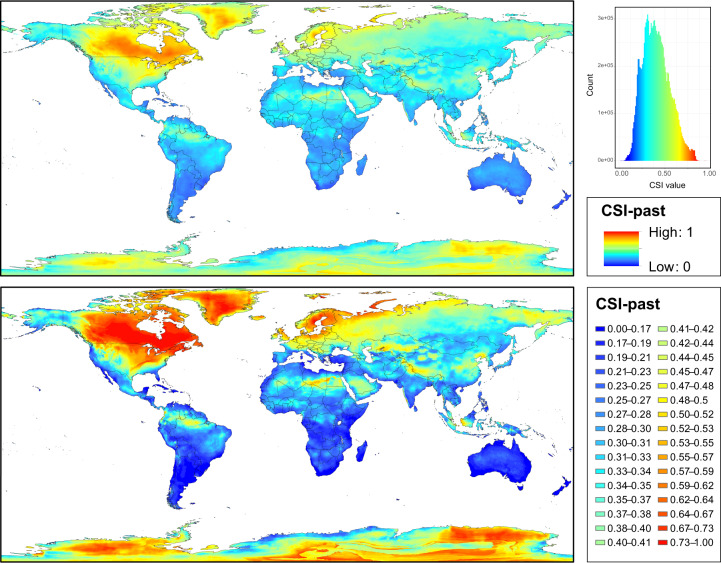
Maps of Climate Stability Index (CSI) values for the past map set from Pliocene (3.3 Ma) to present (1979–2013), at 2.5 arc-min grid resolution. Colours range from blue for low standard deviation (SD) values, which represents areas with low climatic fluctuations (i.e, low values of CSI) during the period Pliocene–present, to red for high SD values, which shows areas where high climatic fluctuations would have taken place (i.e., high values of CSI). On the upper map, the colour ramp shows equal interval breaks. The histogram with frequency and distribution of CSI values is also shown. On the lower map, the colour ramp has been manually adjusted to a defined set of break values (see details in the text).

**Fig. 3:**
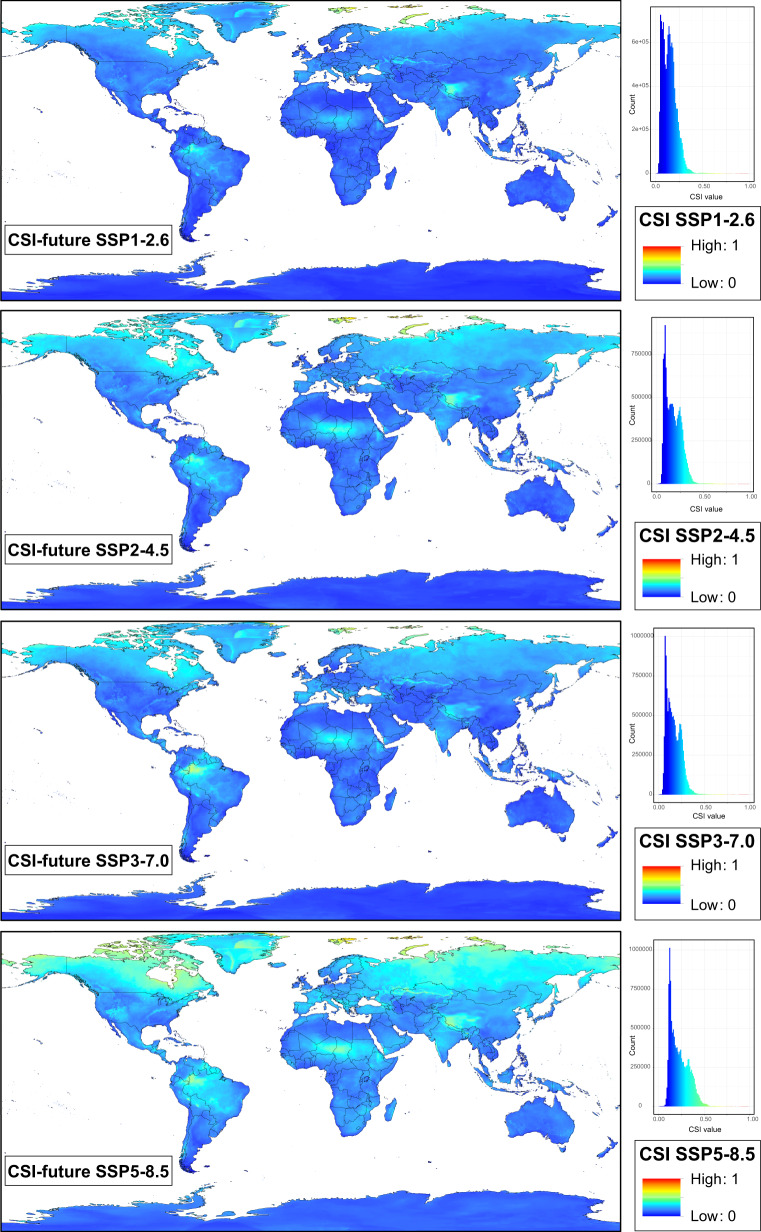
Maps of Climate Stability Index (CSI) values for the future conditions (Shared Socioeconomic Pathways: SSP1-2.6, SSP2-4.5, SSP3-7.0, and SSP5-8.5) from present (1970–2000) to future (2100), at 2.5 arc-min grid resolution. Colours range from blue for low standard deviation (SD) values, which represents areas with low climatic fluctuations (i.e, low values of CSI) from present to future, to red for high SD values, which shows areas where high climatic fluctuations would have taken place (i.e., high values of CSI). The colour ramp shows equal interval breaks. The histogram with frequency and distribution of CSI values is also shown for each future scenario.

**Fig. 4:**
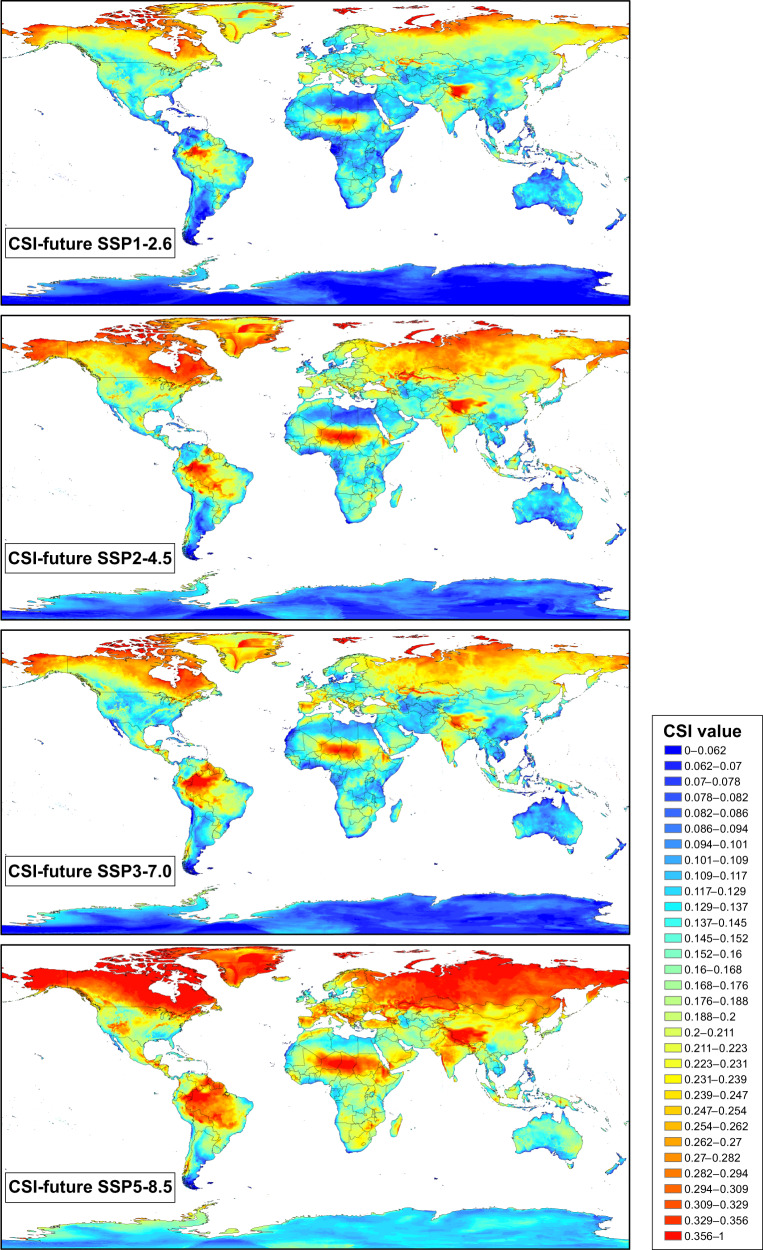
Maps of Climate Stability Index (CSI) values for the future conditions (Shared Socioeconomic Pathways: SSP1-2.6, SSP2-4.5, SSP3-7.0, and SSP5-8.5) from present (1970–2000) to future (2100), at 2.5 arc-min grid resolution. Colours range from blue for low standard deviation (SD) values, which represents areas with low climatic fluctuations (i.e, low values of CSI) from present to future, to red for high SD values, which shows areas where high climatic fluctuations would have taken place (i.e., high values of CSI). The colour ramp has been manually adjusted to a defined set of break values (see details in the text).

The final CSI maps were obtained by summing the SD values of the variables selected and the subsequent outputs normalized (0 to 1) (Figs. 2–4). Histogram plots were represented with ggplot2 R package^24^ and maps were exported with ArcGIS v.10.2.2 (Esri, Redlands, California, USA 2014). The histograms were computed for these final CSI maps, which represent the frequency and distribution of CSI values. We presented the final CSI maps with two different colour ramp schemes with ArcGIS. The first consisted of defining equal interval breaks from 0 to 1. The second was based on defining 32 categories with different value breaks for past and future map sets according to the value frequency shown by the histogram plot, i.e. the category with the highest CSI values (no. 32) was 0.71–1 in the past map set and 0.356–1 in the future map set.

## Data Records

Our data records are available through Figshare^21^ in format of map raster layers (.TIF format).

Two sets of data records are stored:

1. SD-based maps of individual bioclimatic variables, which contain a set of raster layers (one for each variable): 14 for the case of the past map set (bio2, bio3, bio5, bio6, bio7 are missing) and 19 for the future map sets based on median calculations. These layers contain individual measures of the SD of each variable, which could be independently used according to the user’s study purpose or combined according to the user’s preferences to generate a customized CSI.
2. the five CSI-based maps presented in this study, corresponding to CSI-past, CSI-future SSP1-2.6, CSI-future SSP2-4.5, CSI-future SSP3-7.0, and CSI-future SSP5-8.5.

Data files share a common naming pattern:

For *Past map set* from individual variables and CSI, respectively:

“sd_past_<bioclimatic variable>.tif”

“csi_past.tif”

For *Future map sets* from individual variables and CSI, respectively:

“sd_future_<SSP scenario>_<bioclimatic variable>.tif”

“csi_future_<SSP scenario>.tif”

In addition, for the CSI-past map set, we include a map showing the areas affected by sea-level rising periods (intergl_affected.tif) and the raster map used to remove regions with landmasses currently under the sea but that were once emerged (lgm_del_mask.tif; see Supplementary Fig. 1).

## Technical Validation

**Fig. 5:**
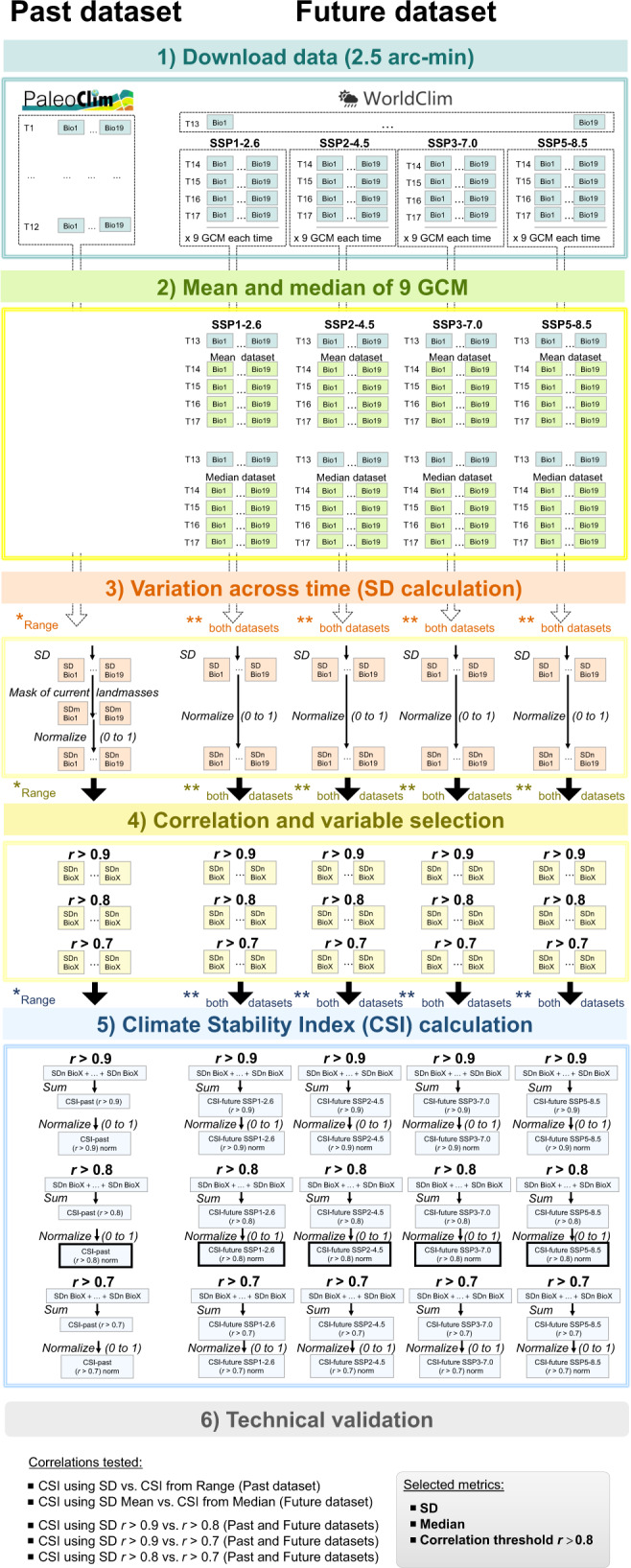
Detailed workflow of analyses employed to calculate and test the robustness of the Climate Stability Index (CSI) for past and future map sets. Final maps presented were obtained with conditions marked with wide-lined frame in the fifth step, which are: SD applying *r* > 0.8 threshold for variable correlation in past map set, and SD from median of nine GCM and applying *r* > 0.8 threshold for variable correlation in future map sets. Note that in the third step further analyses are repeated for the range statistic in the case of past map set and for both future map sets (mean and median).

**Fig. 6:**
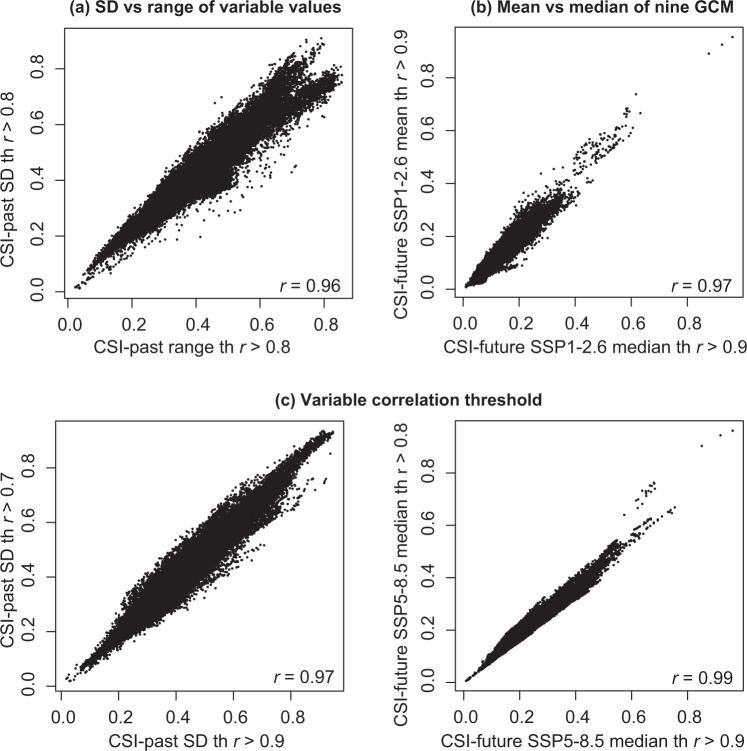
Pearson’s correlation coefficient (*r*) for examples of Climate Stability Index (CSI) pair comparisons: (**a**) between SD and range variable values of the past map set; (**b**) between mean and median of General Circulation Models of future map sets; and (**c**) between *r* > 0.7 and *r* > 0.9 (left) and between *r* > 0.8 and *r* > 0.9 (right) of Pearson cut-off values to remove highly correlated variables.

To evaluate the robustness of the CSI index, we compared its performance by varying the statistics used for its calculation (see detailed workflow in Fig. 5). For the past map set, the CSI was computed independently by means of SD and range. For the future map sets, the CSI was computed using the mean and the median of the nine GCMs to obtain individual future rasters for each time and SSPs. In parallel, the sensitivity derived from selecting a more restrictive variable correlation threshold (*r* > 0.7), an intermediate (*r* > 0.8), or a less restrictive cut-off value (*r* > 0.9) was also checked. In Supplementary Tables 1 and 2 the variables selected for each *r* threshold tested are specified. All pairs of CSI rasters were compared through Pearson’s correlation analyses, which showed a very high *r* value, ranging from 0.93 to 1.00. All *r* values in Table 3 and all correlation scatterplots are available in Supplementary Fig. 2 and some examples are included in Fig. 6. As a conclusion, methodological choices resulted in a non-remarkable impact of metric selected (mean vs. median, SD vs. range) or correlation threshold set (*r* > 0.7, 0.8, 0.9). Our choice was using the SD, median, and threshold *r* > 0.8, to draw the CSI-based maps.

**Table 3:** Correlation values (Pearson’s *r*) of Technical Validation procedure.

Validation category	Correlation pairs	*r*
SD vs. range	CSI-past SD th 0.7 vs. CSI-past range th 0.7	0.94
	CSI-past SD th 0.8 vs. CSI-past range th 0.8	0.96
	CSI-past SD th 0.9 vs. CSI-past range th 0.9	0.97
mean vs. median	CSI-future mean SSP1-2.6 th 0.7 vs. CSI-future median SSP1-2.6 th 0.7	1.00
	CSI-future mean SSP1-2.6 th 0.8 vs. CSI-future median SSP1-2.6 th 0.8	1.00
	CSI-future mean SSP1-2.6 th 0.9 vs. CSI-future median SSP1-2.6 th 0.9	0.97
	CSI-future mean SSP2-4.5 th 0.7 vs. CSI-future median SSP2-4.5 th 0.7	1.00
	CSI-future mean SSP2-4.5 th 0.8 vs. CSI-future median SSP2-4.5 th 0.8	1.00
	CSI-future mean SSP2-4.5 th 0.9 vs. CSI-future median SSP2-4.5 th 0.9	1.00
	CSI-future mean SSP3-7.0 th 0.7 vs. CSI-future median SSP3-7.0 th 0.7	1.00
	CSI-future mean SSP3-7.0 th 0.8 vs. CSI-future median SSP3-7.0 th 0.8	1.00
	CSI-future mean SSP3-7.0 th 0.9 vs. CSI-future median SSP3-7.0 th 0.9	1.00
	CSI-future mean SSP5-8.5 th 0.7 vs. CSI-future median SSP5-8.5 th 0.7	1.00
	CSI-future mean SSP5-8.5 th 0.8 vs. CSI-future median SSP5-8.5 th 0.8	1.00
	CSI-future mean SSP5-8.5 th 0.9 vs. CSI-future median SSP5-8.5 th 0.9	1.00
threshold (*r*)	CSI-past SD th 0.7 vs. CSI-past SD th 0.8	0.95
	CSI-past SD th 0.7 vs. CSI-past SD th 0.9	0.97
	CSI-past SD th 0.8 vs. CSI-past SD th 0.9	0.98
	CSI-past range th 0.7 vs. CSI-past range th 0.8	0.98
	CSI-past range th 0.7 vs. CSI-past range th 0.9	0.95
	CSI-past range th 0.8 vs. CSI-past range th 0.9	0.99
	CSI-future mean SSP1-2.6 th 0.7 vs. CSI-future mean SSP1-2.6 th 0.8	0.99
	CSI-future mean SSP1-2.6 th 0.7 vs. CSI-future mean SSP1-2.6 th 0.9	0.99
	CSI-future mean SSP1-2.6 th 0.8 vs. CSI-future mean SSP1-2.6 th 0.9	0.99
	CSI-future mean SSP2-4.5 th 0.7 vs. CSI-future mean SSP2-4.5 th 0.8	0.96
	CSI-future mean SSP2-4.5 th 0.7 vs. CSI-future mean SSP2-4.5 th 0.9	0.93
	CSI-future mean SSP2-4.5 th 0.8 vs. CSI-future mean SSP2-4.5 th 0.9	0.99
	CSI-future mean SSP3-7.0 th 0.7 vs. CSI-future mean SSP3-7.0 th 0.8	0.98
	CSI-future mean SSP3-7.0 th 0.7 vs. CSI-future mean SSP3-7.0 th 0.9	0.99
	CSI-future mean SSP3-7.0 th 0.8 vs. CSI-future mean SSP3-7.0 th 0.9	0.98
	CSI-future mean SSP5-8.5 th 0.7 vs. CSI-future mean SSP5-8.5 th 0.8	0.98
	CSI-future mean SSP5-8.5 th 0.7 vs. CSI-future mean SSP5-8.5 th 0.9	0.99
	CSI-future mean SSP5-8.5 th 0.8 vs. CSI-future mean SSP5-8.5 th 0.9	0.99
	CSI-future median SSP1-2.6 th 0.7 vs. CSI-future median SSP1-2.6 th 0.8	0.99
	CSI-future median SSP1-2.6 th 0.7 vs. CSI-future median SSP1-2.6 th 0.9	0.96
	CSI-future median SSP1-2.6 th 0.8 vs. CSI-future median SSP1-2.6 th 0.9	0.96
	CSI-future median SSP2-4.5 th 0.7 vs. CSI-future median SSP2-4.5 th 0.8	0.96
	CSI-future median SSP2-4.5 th 0.7 vs. CSI-future median SSP2-4.5 th 0.9	0.93
	CSI-future median SSP2-4.5 th 0.8 vs. CSI-future median SSP2-4.5 th 0.9	0.99
	CSI-future median SSP3-7.0 th 0.7 vs. CSI-future median SSP3-7.0 th 0.8	0.98
	CSI-future median SSP3-7.0 th 0.7 vs. CSI-future median SSP3-7.0 th 0.9	0.99
	CSI-future median SSP3-7.0 th 0.8 vs. CSI-future median SSP3-7.0 th 0.9	0.98
	CSI-future median SSP5-8.5 th 0.7 vs. CSI-future median SSP5-8.5 th 0.8	0.98
	CSI-future median SSP5-8.5 th 0.7 vs. CSI-future median SSP5-8.5 th 0.9	0.99
	CSI-future median SSP5-8.5 th 0.8 vs. CSI-future median SSP5-8.5 th 0.9	0.99

Abbreviations used: SD (Standard Deviation), CSI (Climate Stability Index), th (threshold), *r* (*r* statistic of Pearson’s correlation analysis).

Before concluding, however, it should be acknowledged that the fact of not using the same approaches for past and future climate stability assessments introduces a degree of uncertainty when considered as a whole. To obtain CSI-past maps we are using *PaleoClim*, which provide simulations from single models for different time periods, whereas for CSI-future maps we are employing an ensemble of nine model simulations (and, thus, there is regional variability in stability).

## Supplementary information

Supplementary Table 1

Supplementary Table 2

Supplementary Figures

## Data Availability

Commented R codes used to generate CSI are available at Figshare^21^.
