# Supplementary material for: Climate Stability Index maps, a global high resolution cartography of climate stability from Pliocene to 2100
Source: Sci Data. 2022 Feb 10;9:48. doi: 10.1038/s41597-022-01144-5 (PMC8831633; doi:10.1038/s41597-022-01144-5)
Supplement: Supplementary file 1 — Supplementary Table 1 [file 41597_2022_1144_MOESM1_ESM.docx]

**Supplementary Table 1.** Bioclimatic variables used to generate the Climate Stability Index (CSI), considering the median calculation of nine future scenarios, for each analysis of different *r* (*r* statistic of Pearson’s correlation analysis; *r* > 0.9, *r* > 0.8, *r* > 0.7). For each variable we include the following information: the units, whether they are included on a climatic map set to calculate the CSI. Note that bio2, bio3, bio5, bio6 and bio7 were not included for the calculation of CSI past, as these variables are not available in *PaleoClim* database^1^ for T1 (M2, Pliocene, ca. 3.3 Ma), T2 (mid-Pliocene warm period, Pliocene, 3.205 Ma), and T3 (MIS19, Pleistocene, ca. 787 ka). Unit abbreviations: °C (Celsius), mm (millimetres), dls (dimensionless).

| Variable | Unit | Included in CSI(*r* > 0.9) | Included in CSI(*r* > 0.8) | Included in CSI(*r* > 0.7) |
| --- | --- | --- | --- | --- |
| Bio1: Annual mean temperature | °C | Future (SSP2) | PastFuture (SSP3) | Future (SSP2, SSP5) |
| Bio2: Mean Diurnal Range (Mean of monthly (max temp - min temp)) | °C | Future (SSP1, SSP2, SSP3, SSP5) | Future (SSP1, SSP2, SSP3, SSP5) | Future (SSP1, SSP2, SSP3, SSP5) |
| Bio3: Isothermality (BIO2/BIO7) (×100) | dls | Future (SSP1, SSP2, SSP3, SSP5) | Future (SSP1, SSP2, SSP3, SSP5) | Future (SSP1, SSP2, SSP3, SSP5) |
| Bio4: Temperature Seasonality (standard deviation ×100) | °C | PastFuture (SSP2, SSP5) | Past | Past Future (SSP1) |
| Bio5: Max Temperature of Warmest Month | °C | Future (SSP1, SSP2, SSP3) | Future (SSP3) | Future (SSP1, SSP3) |
| Bio6: Min Temperature of Coldest Month | °C | Future (SSP3) | Future (SSP1, SSP2) |  |
| Bio7: Temperature Annual Range (BIO5-BIO6) | °C | Future (SSP1, SSP3) | Future (SSP1, SSP5) | Future (SSP3) |
| Bio8: Mean Temperature of Wettest Quarter | °C | Past Future (SSP1, SSP2, SSP3, SSP5) | Future (SSP1, SSP2, SSP3, SSP5) | Past Future (SSP1, SSP2, SSP3, SSP5) |
| Bio9: Mean Temperature of Driest Quarter | °C | Past Future (SSP1, SSP2, SSP3, SSP5) | PastFuture (SSP1, SSP2, SSP3, SSP5) | Past Future (SSP1, SSP2, SSP3, SSP5) |
| Bio10: Mean Temperature of Warmest Quarter | °C | PastFuture (SSP3, SSP5) | Future (SSP1, SSP2, SSP5) | Future (SSP2, SSP5) |
| Bio11: Mean Temperature of Coldest Quarter | °C | Past Future (SSP1, SSP5) | Past |  |
| Bio12: Annual Precipitation | mm | Past Future (SSP1, SSP2, SSP3, SSP5) | PastFuture (SSP1, SSP3, SSP5) | Past Future (SSP1) |
| Bio13: Precipitation of Wettest Month | mm | Future (SSP2, SSP3) | PastFuture (SSP3, SSP5) | Future (SSP3) |
| Bio14: Precipitation of Driest Month | mm | Future (SSP5) | PastFuture (SSP1) | Future (SSP1, SSP5) |
| Bio15: Precipitation Seasonality (Coefficient of Variation) | dls | Past Future (SSP1, SSP2, SSP3, SSP5) | PastFuture (SSP1, SSP2, SSP3, SSP5) | Past Future (SSP1, SSP2, SSP3, SSP5) |
| Bio16: Precipitation of Wettest Quarter | mm | Past Future (SSP1, SSP5) | Future (SSP2) | PastFuture (SSP2, SSP5) |
| Bio17: Precipitation of Driest Quarter | mm | Past Future (SSP1, SSP2, SSP3) | Future (SSP2, SSP3, SSP5) | PastFuture (SSP2, SSP3) |
| Bio18: Precipitation of Warmest Quarter | mm | Past Future (SSP1, SSP2, SSP3, SSP5) | PastFuture (SSP1, SSP2, SSP3, SSP5) | Past Future (SSP1, SSP2, SSP3, SSP5) |
| Bio19: Precipitation of Coldest Quarter | mm | Past Future (SSP1, SSP2, SSP3, SSP5) | PastFuture (SSP1, SSP2, SSP3, SSP5) | Past Future (SSP1, SSP2, SSP3, SSP5) |

^1^ Brown, J. L., Hill, D. J., Dolan, A. M., Carnaval, A. C. & Haywood, A. M. PaleoClim, high spatial resolution paleoclimate surfaces for global land areas. *Sci. Data* **5**, 180254, https://doi.org/10.1038/sdata.2018.254 (2018).
