# Supplementary material for: Climate Stability Index maps, a global high resolution cartography of climate stability from Pliocene to 2100
Source: Sci Data. 2022 Feb 10;9:48. doi: 10.1038/s41597-022-01144-5 (PMC8831633; doi:10.1038/s41597-022-01144-5)
Supplement: Supplementary file 3 — Supplementary Figures [file 41597_2022_1144_MOESM3_ESM.pdf]

Data Descriptor: Climate Stability Index maps, a global high resolution cartography of climate stability from Pliocene to 2100

Sonia Herrando-Moraira, Neus Nualart, Mercè Galbany-Casals, Núria Garcia-Jacas, Haruka Ohashi, Tetsuya Matsui, Alfonso Susanna, Cindy Q. Tang & Jordi López-Pujol

Table of contents

Supplementary Fig. 1 ……………………………………………….........……. page 2
Supplementary Fig. 2 ……………………………………………………………... pages 3–25

Supplementary Fig. 1 (**a**) Map of Climate Stability Index (CSI) values for the past map set [Pliocene (3.3 Ma) to present (1979–2013)], at 2.5 arc-min grid resolution. All pixels have been considered in calculations, despite the fact that in some regions where sea-level dropped during cold periods (T1, T3, T5, T6, T7, T8, T9, see Fig. 1 for time period names) there are values for only one or few time periods, while the remaining time periods have NoData codes. (**b**) Map showing the areas (in red) affected by some sea-level rising periods (T2, T4, T10, see Fig. 1 for time period names). A raster layer with the affected areas is available at Figshare[1].

[1] Herrando-Moraira, S. *et al*. Raster_layers_R_scripts.zip. *figshare* https://doi.org/10.6084/m9.figshare.14672637 (2021).

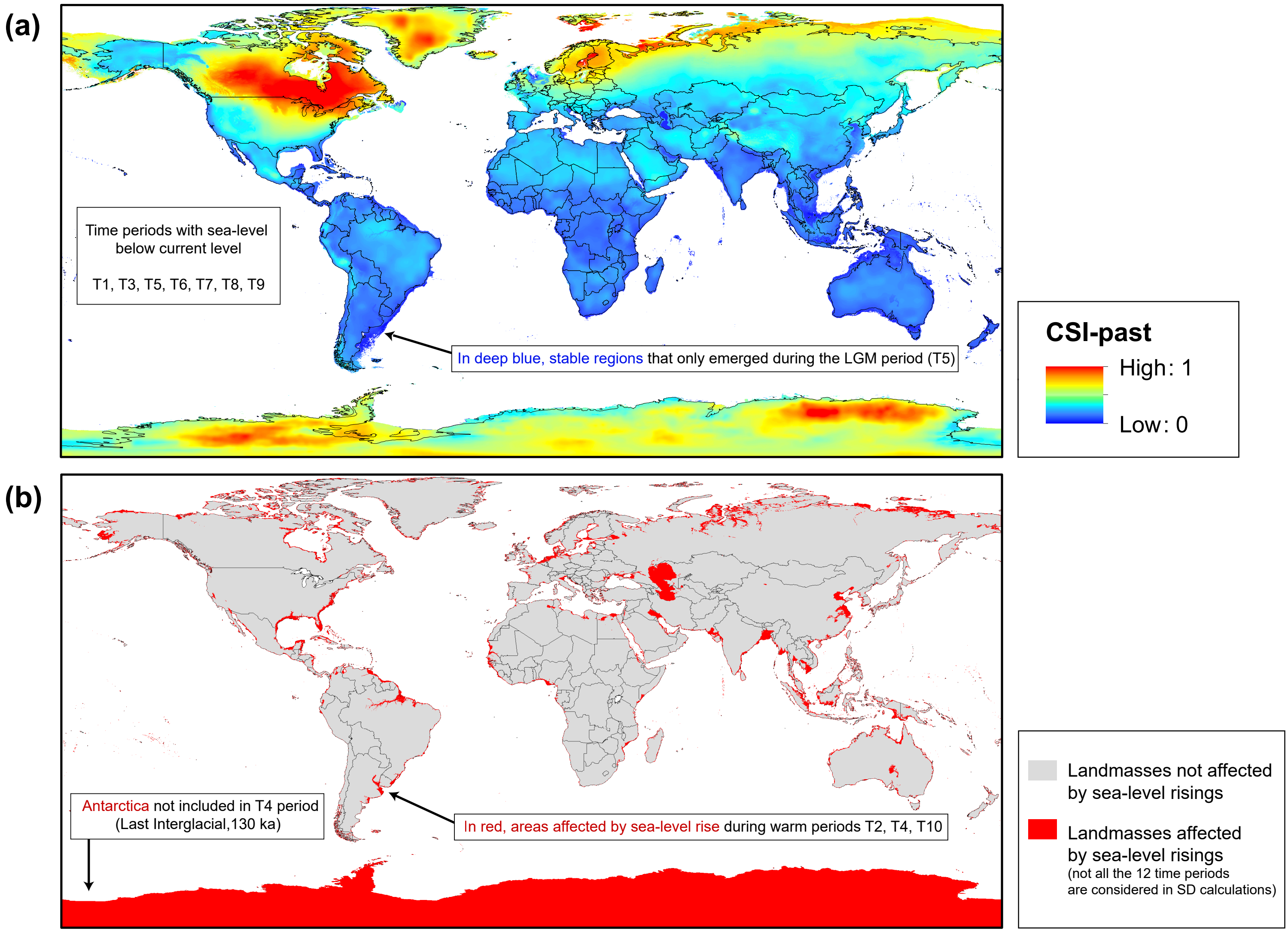

Supplementary Fig. 2 Correlation values (Pearson's *r*) and correlation scatterplots of Technical validation procedure (see Table 3). The graphics follow the same order as in Table 3.

threshold $r = 0.7$ SD vs $r = 0.7$ range

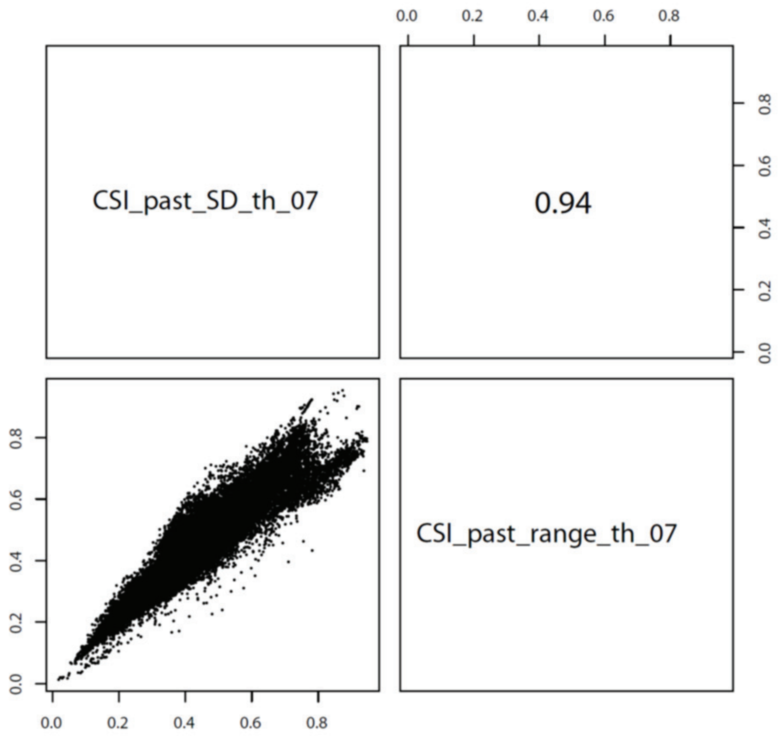

threshold $r = 0.8$ SD vs $r = 0.8$ range

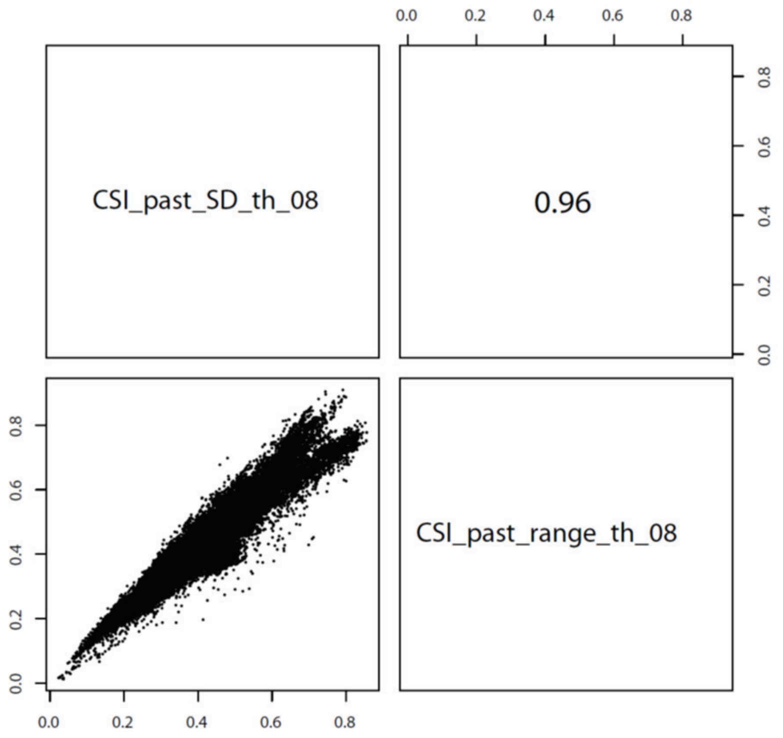

threshold $r = 0.9$ SD vs $r = 0.9$ range

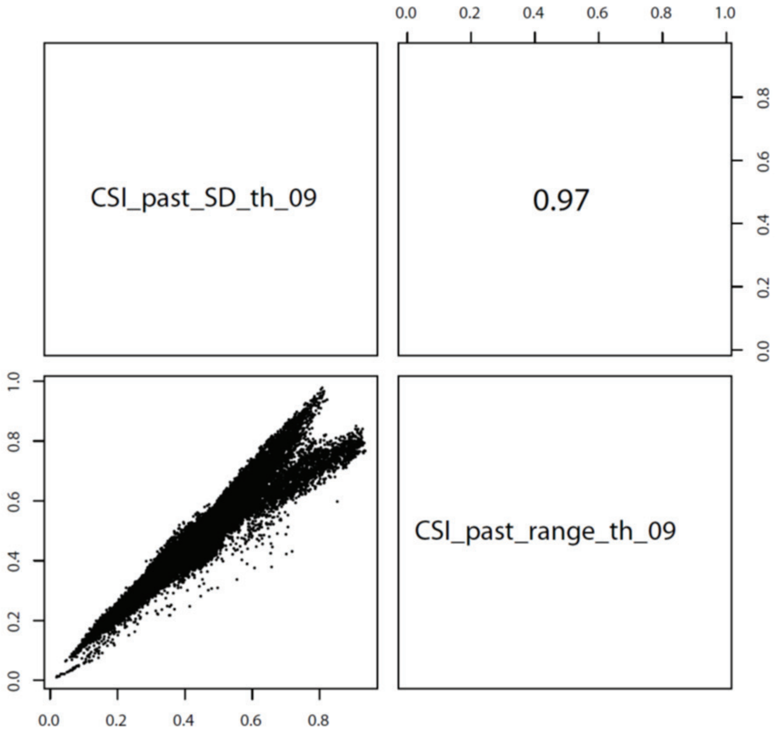

threshold $r = 0.7$ mean vs $r = 0.7$ median

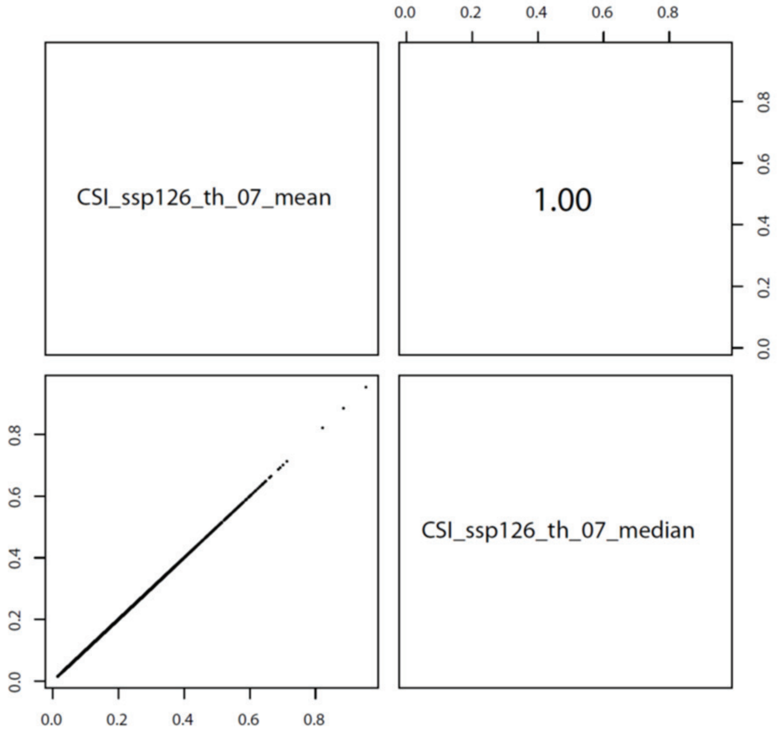

threshold r = 0.8 mean vs r = 0.8 median

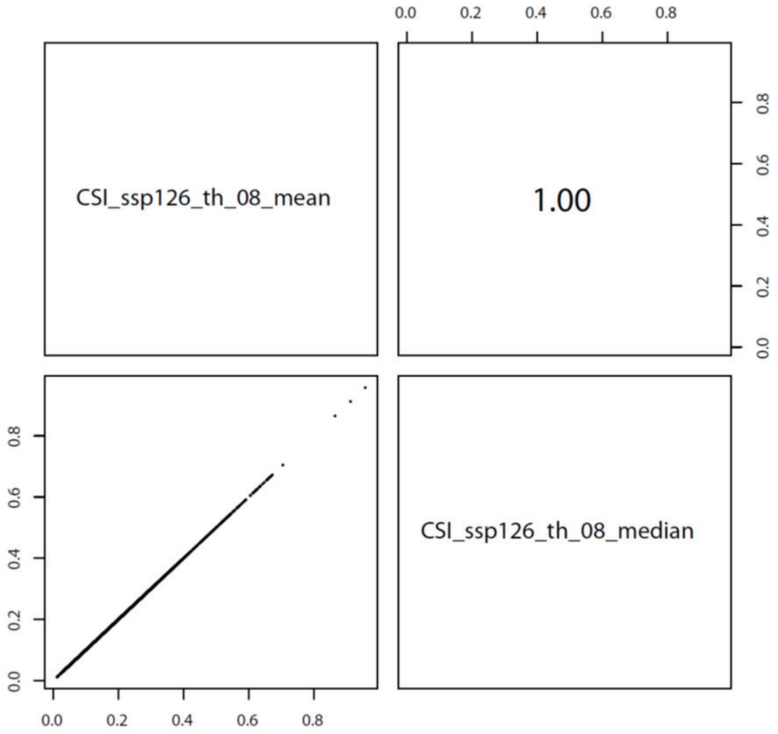

threshold r = 0.9 mean vs r = 0.9 median

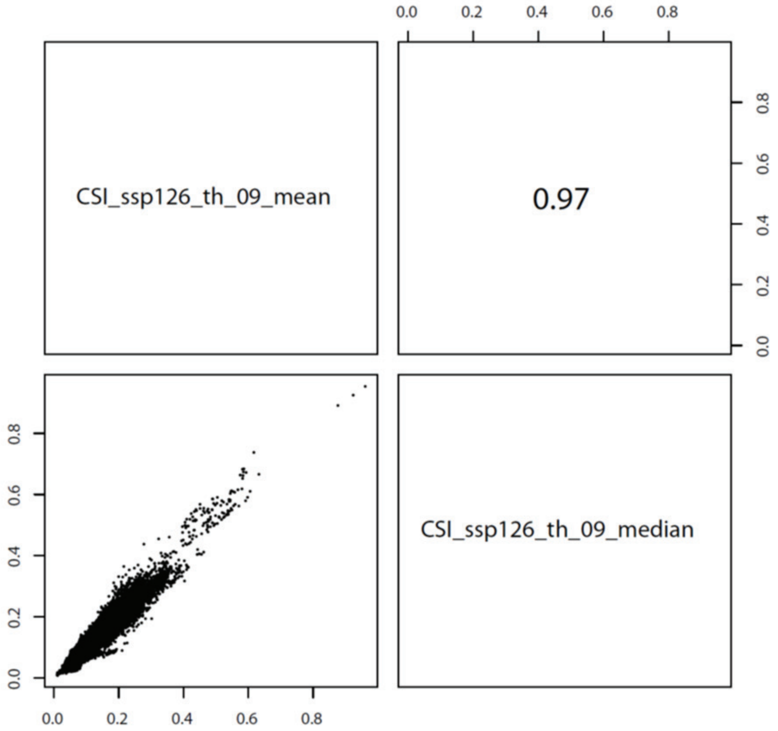

threshold r = 0.7 mean vs r = 0.7 median

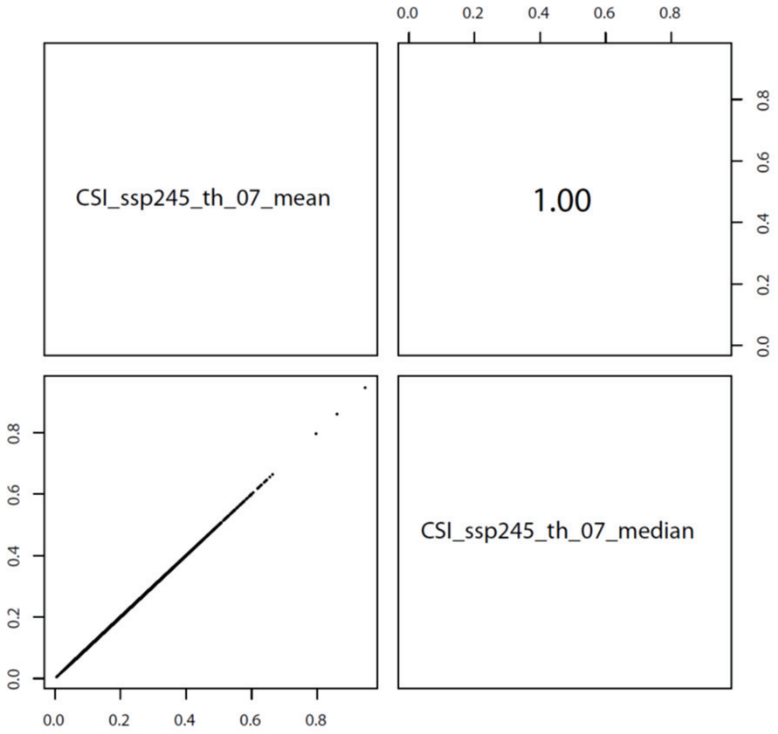

threshold $r = 0.8$ mean vs $r = 0.8$ median

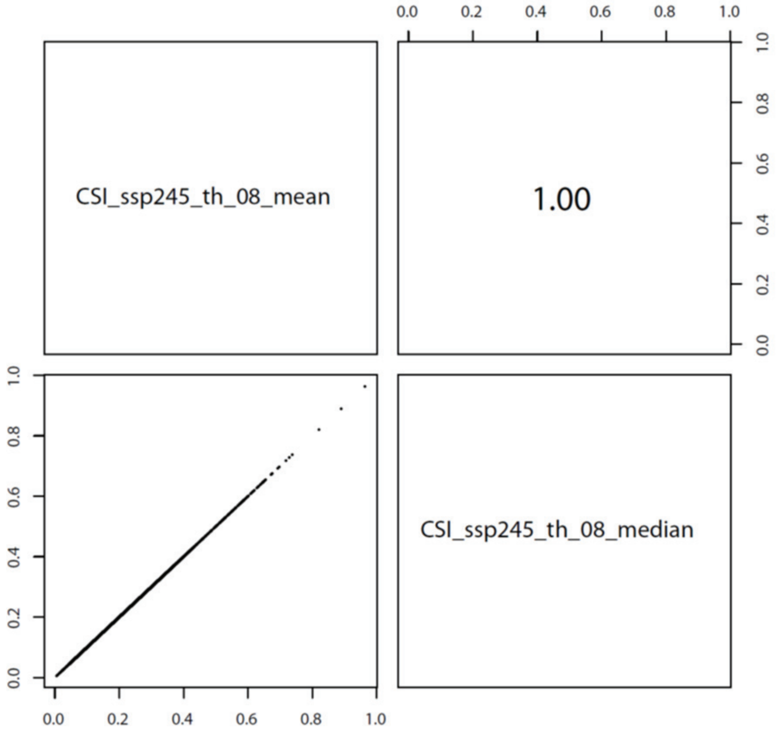

threshold $r = 0.9$ mean vs $r = 0.9$ median

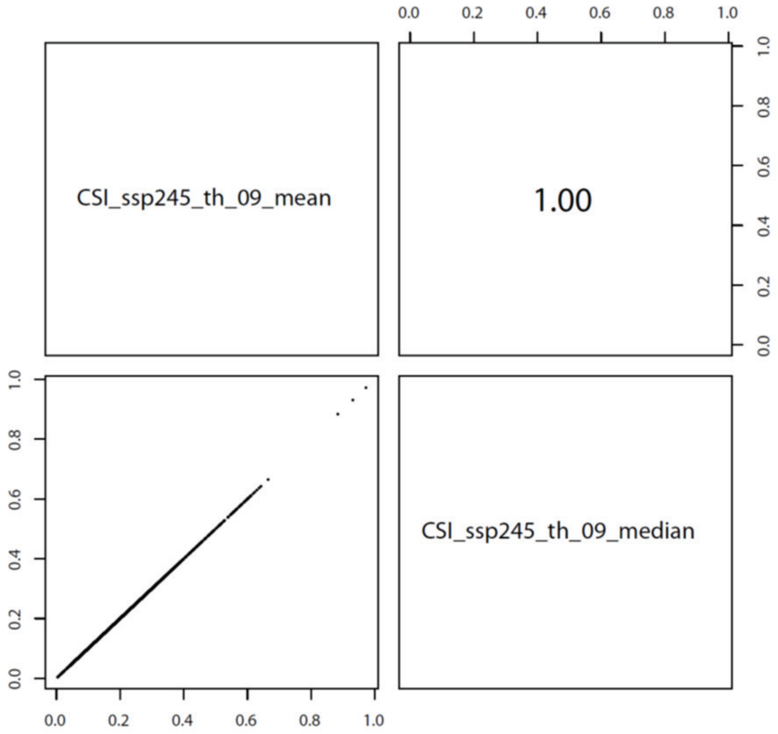

threshold $r = 0.7$ mean vs $r = 0.7$ median

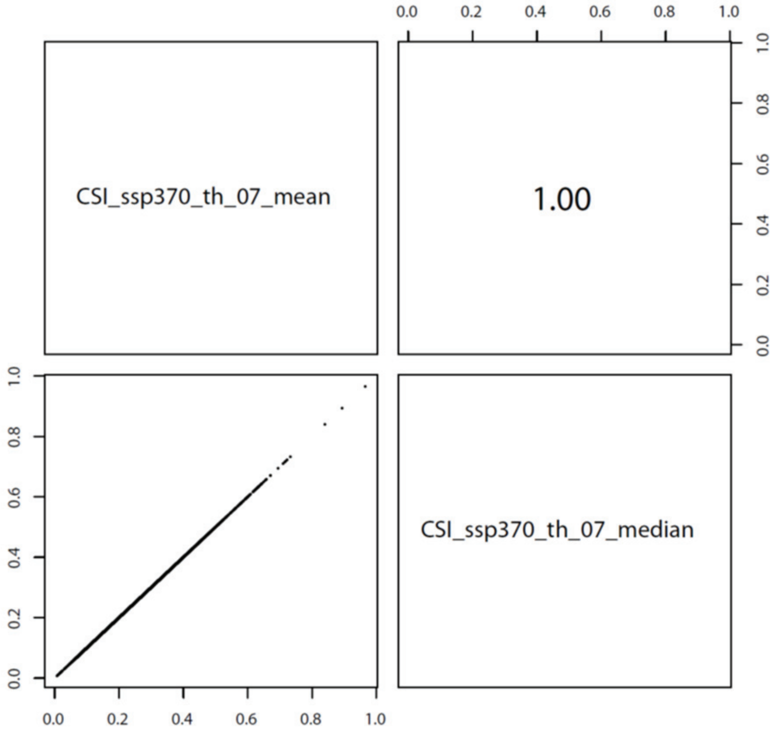

threshold $r = 0.8$ mean vs $r = 0.8$ median

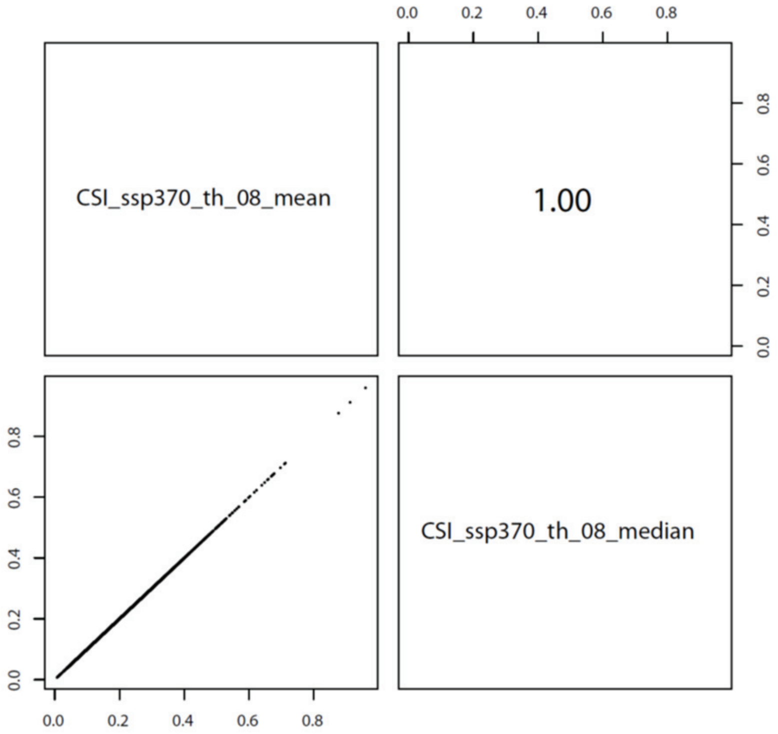

threshold $r = 0.9$ mean vs $r = 0.9$ median

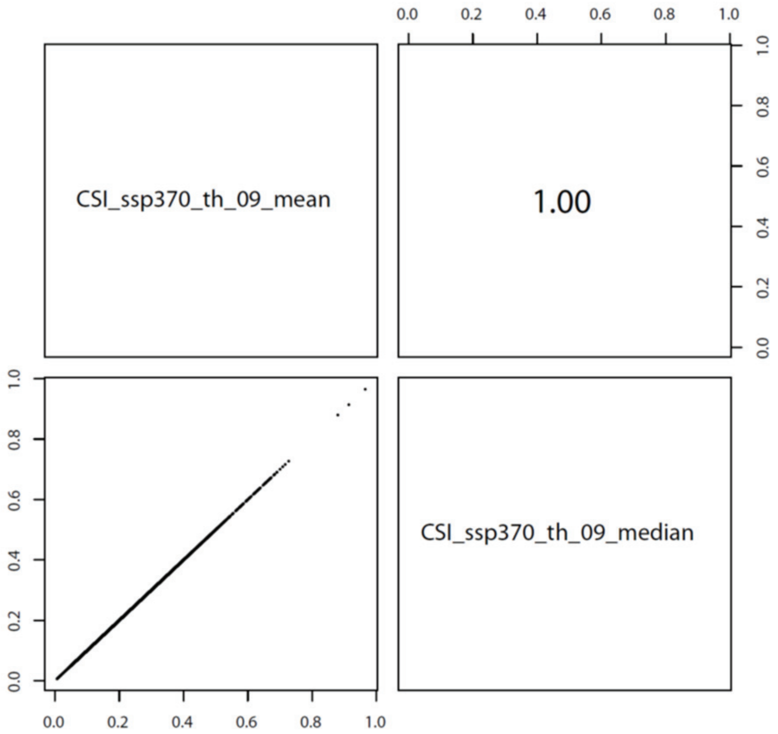

threshold $r = 0.7$ mean vs $r = 0.7$ median

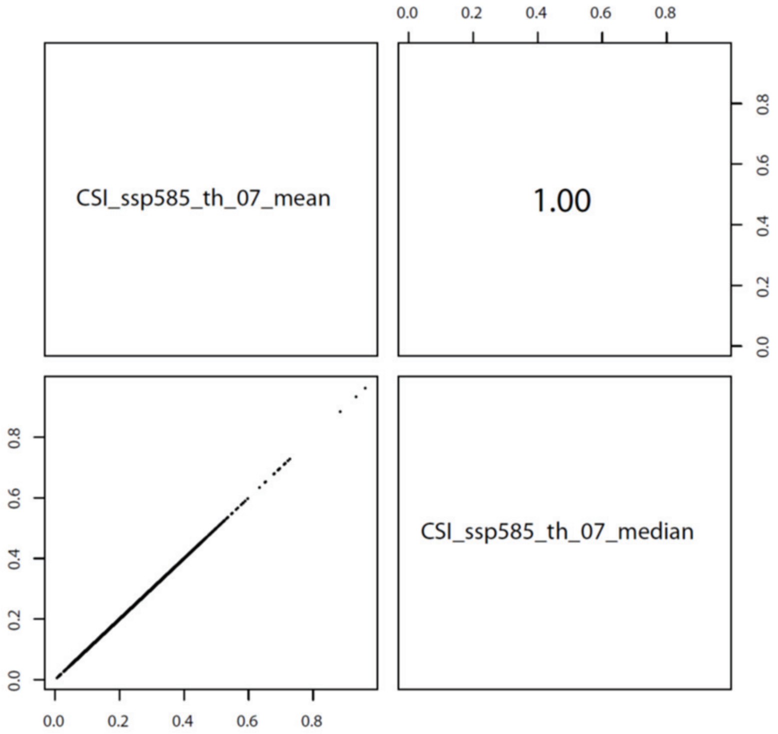

threshold $r = 0.8$ mean vs $r = 0.8$ median

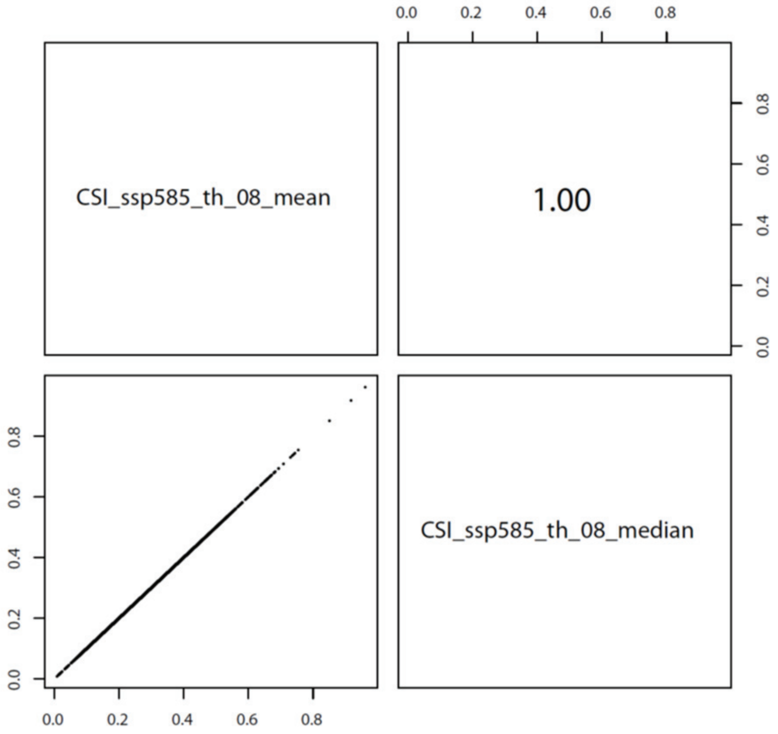

threshold $r = 0.9$ mean vs $r = 0.9$ median

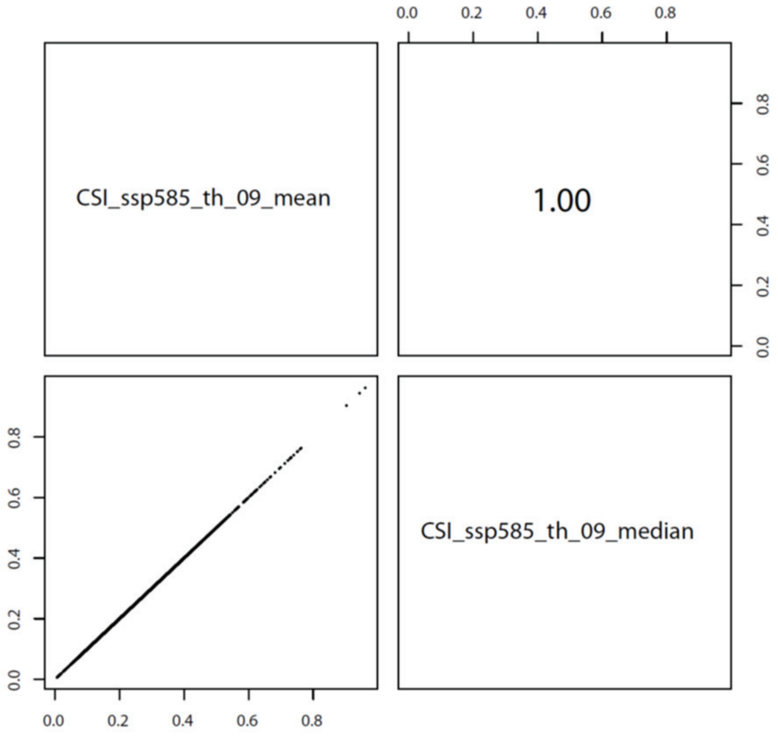

threshold $r = 0.7$ vs $r = 0.8$

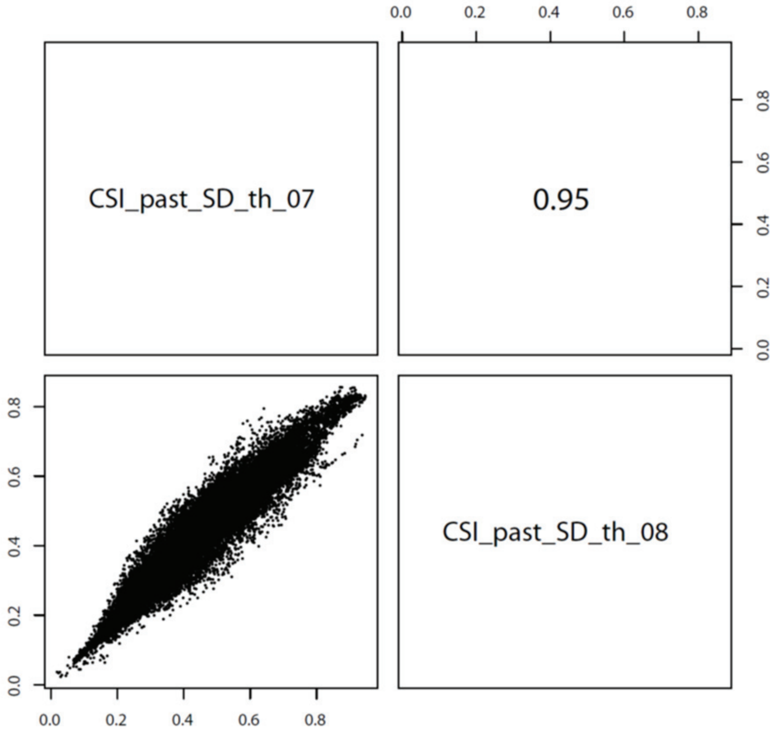

threshold $r = 0.7$ vs $r = 0.9$

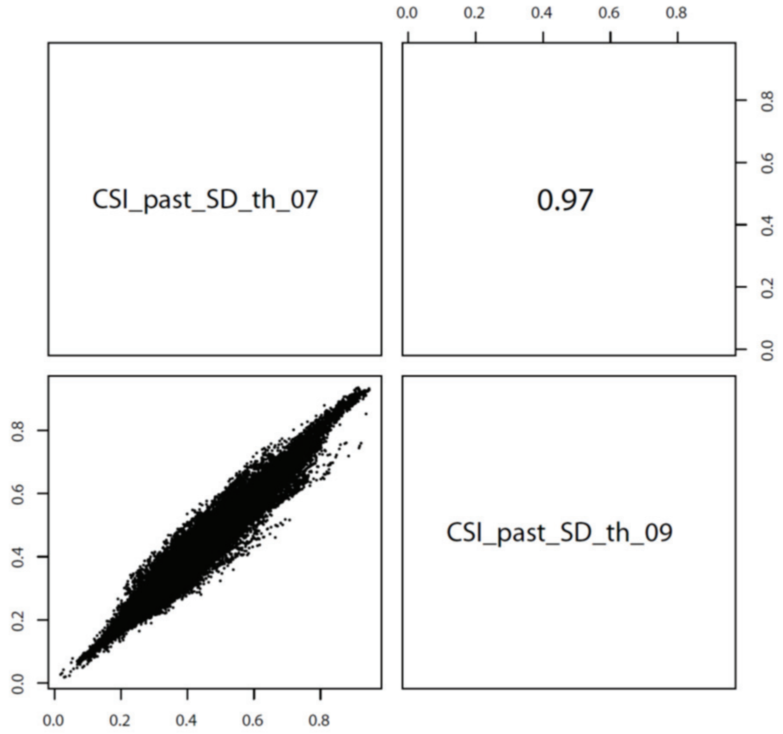

threshold $r = 0.8$ vs $r = 0.9$

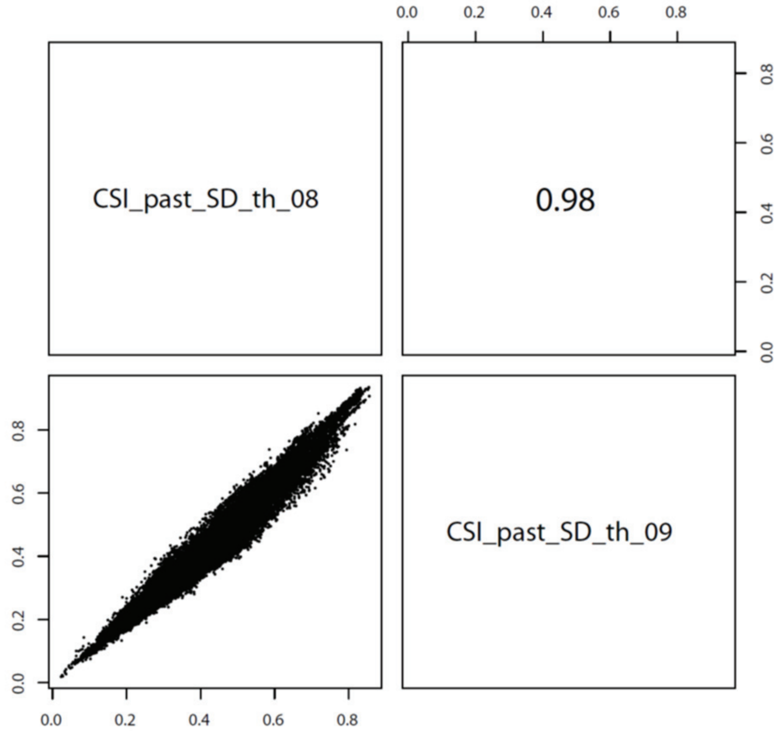

threshold $r = 0.7$ vs $r = 0.8$

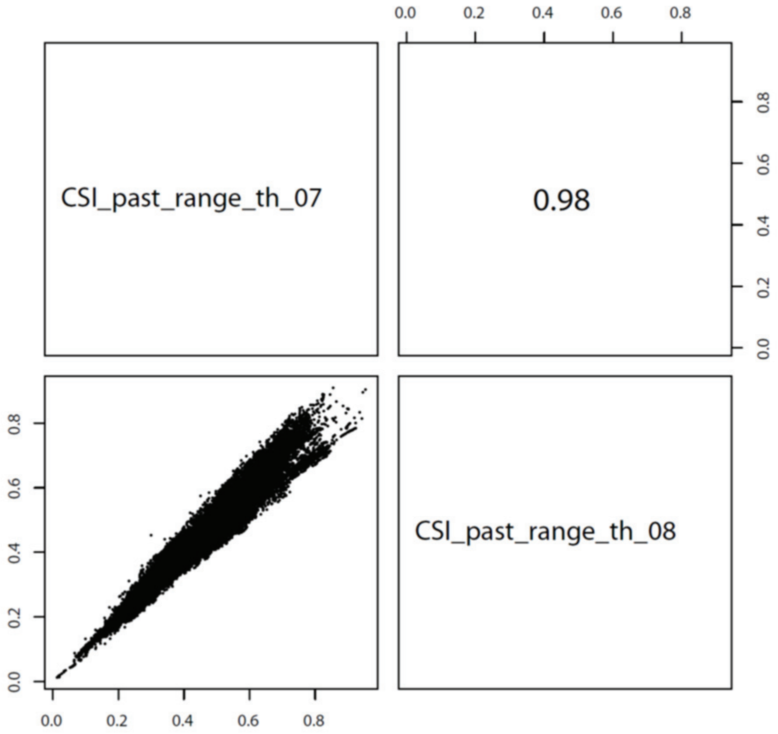

threshold $r = 0.7$ vs $r = 0.9$

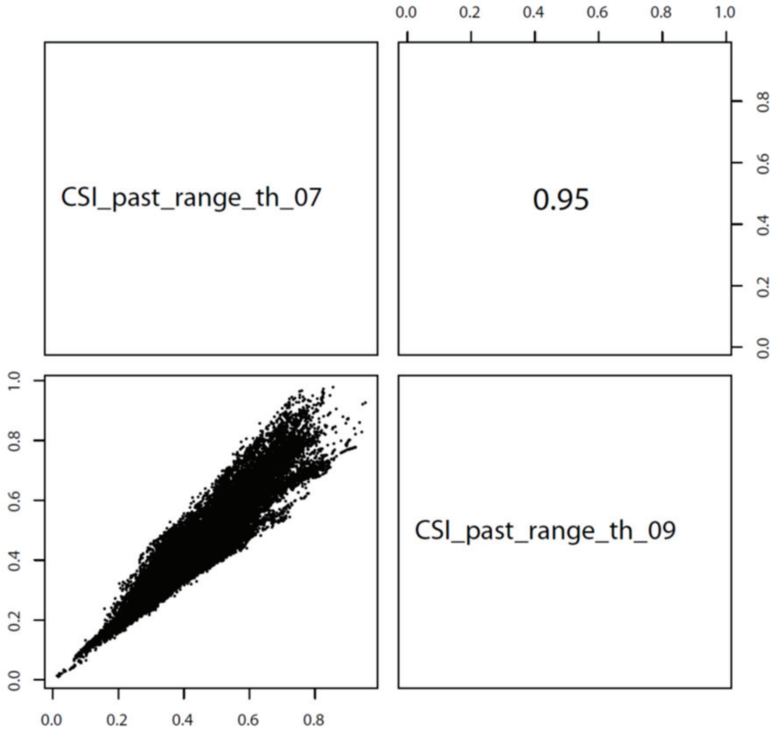

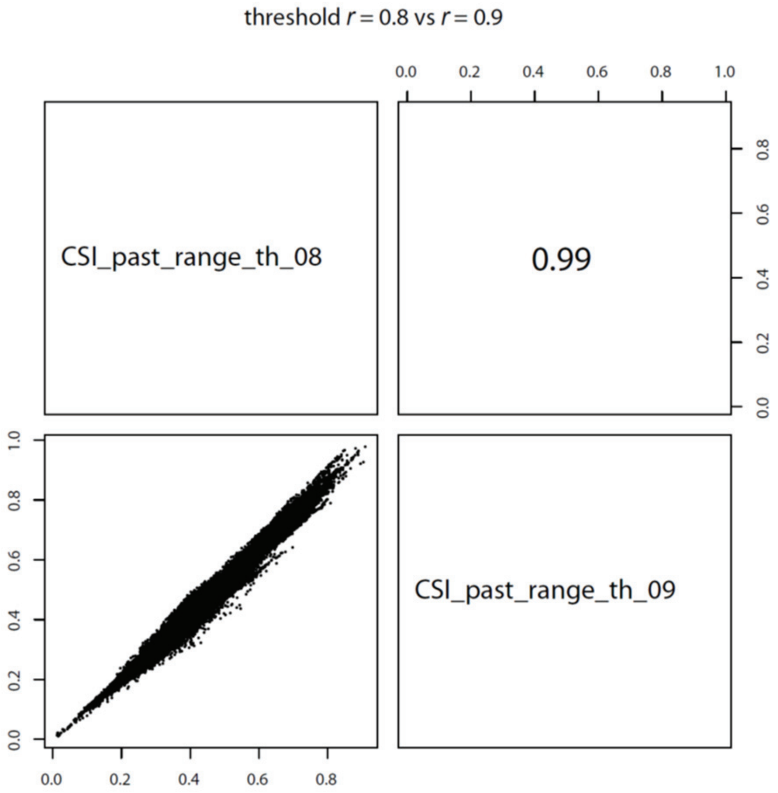
threshold r = 0.8 vs r = 0.9
CSI_past_range_th_08
0.99
CSI_past_range_th_09

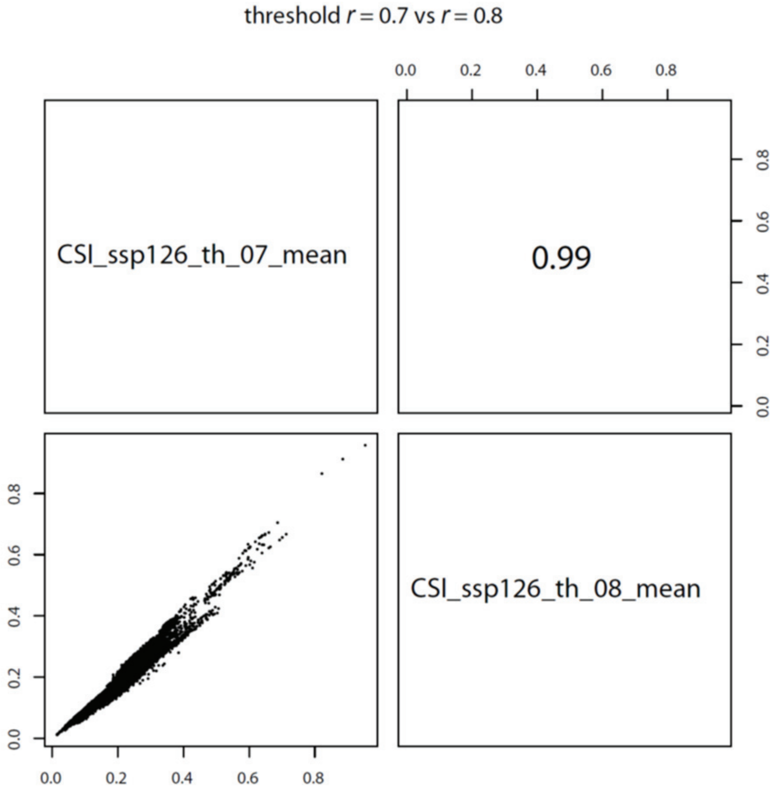
threshold r = 0.7 vs r = 0.8
CSI_ssp126_th_07_mean
0.99
CSI_ssp126_th_08_mean

threshold $r = 0.7$ vs $r = 0.9$

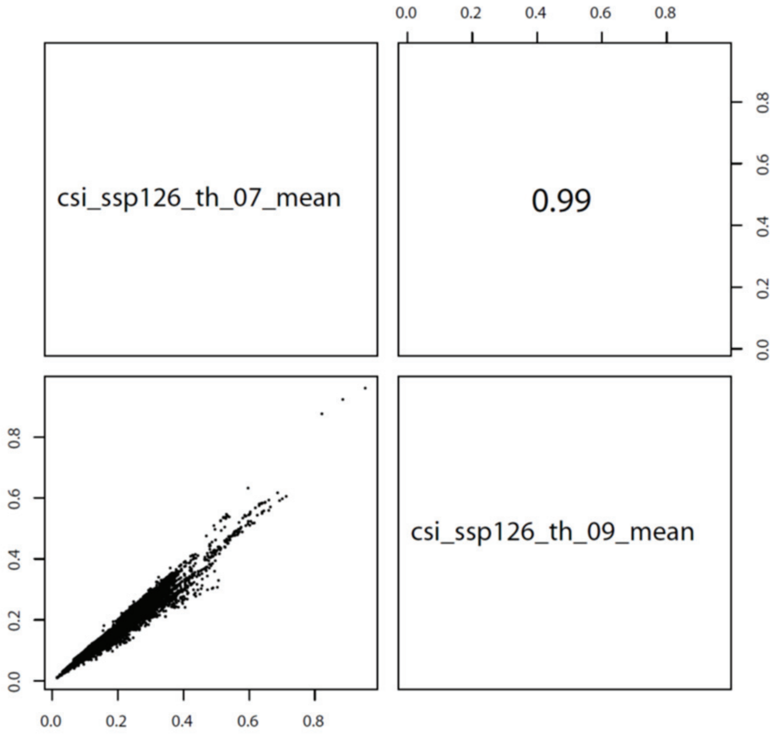

threshold $r = 0.8$ vs $r = 0.9$

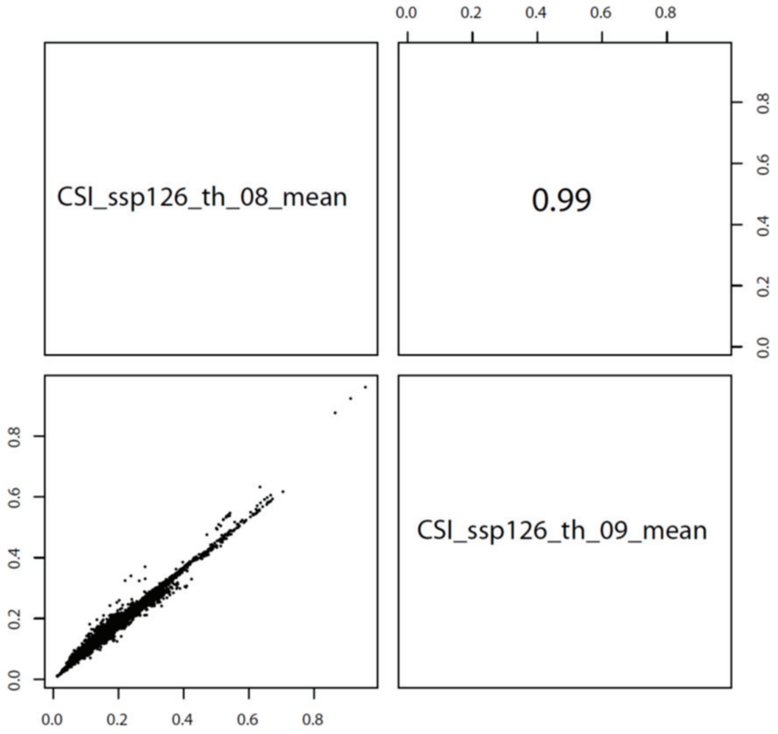

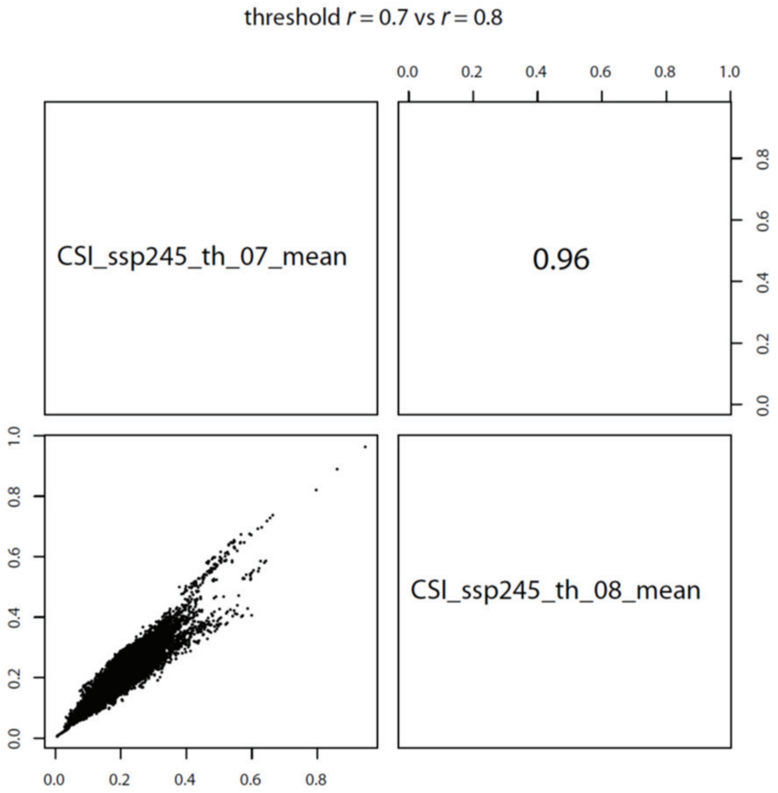
threshold r = 0.7 vs r = 0.8
CSI_ssp245_th_07_mean
0.96
CSI_ssp245_th_08_mean
0.0
0.2
0.4
0.6
0.8
1.0

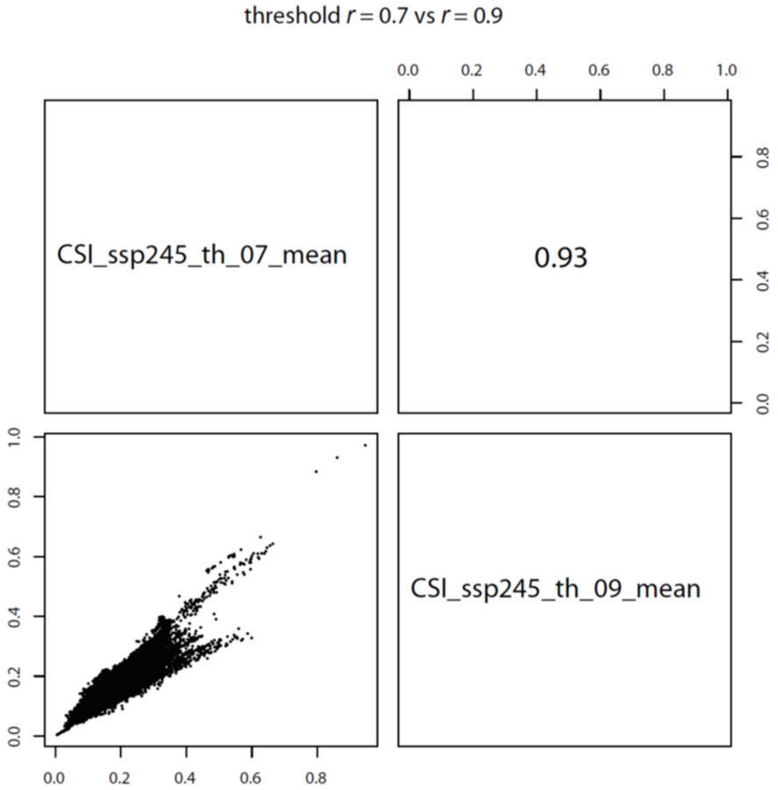
threshold r = 0.7 vs r = 0.9
CSI_ssp245_th_07_mean
0.93
CSI_ssp245_th_09_mean
0.0
0.2
0.4
0.6
0.8
1.0

threshold $r = 0.8$ vs $r = 0.9$

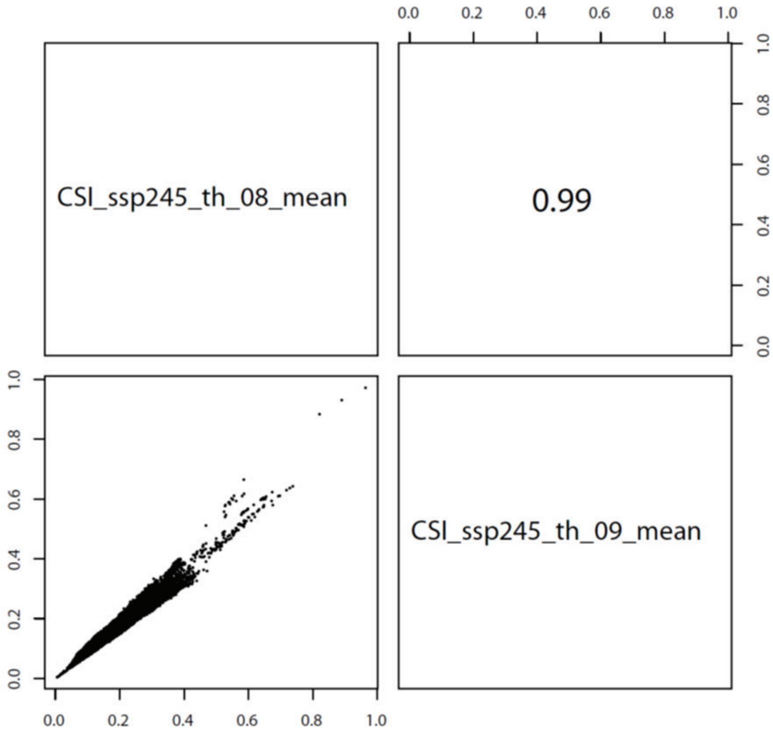

threshold $r = 0.7$ vs $r = 0.8$

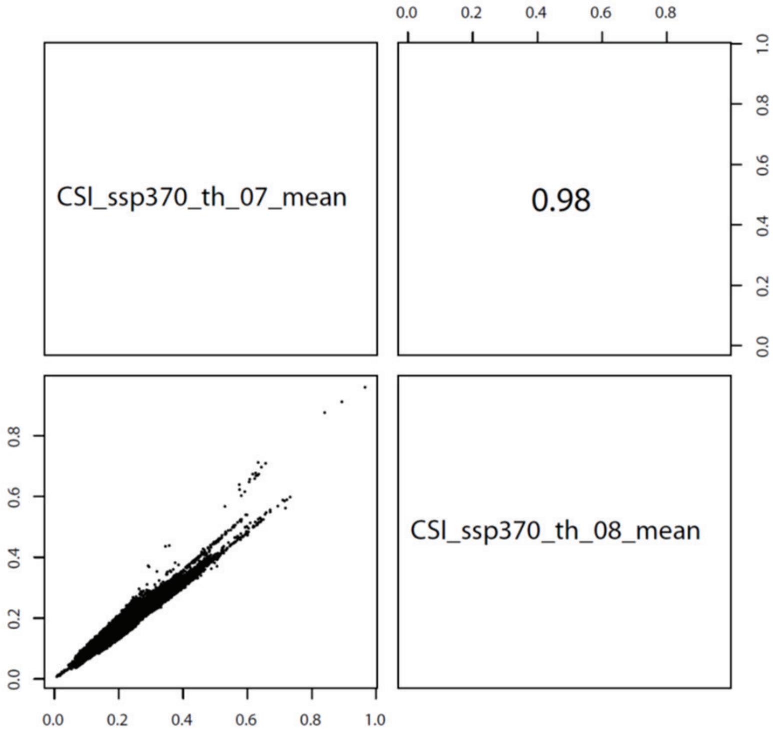

threshold $r = 0.7$ vs $r = 0.9$

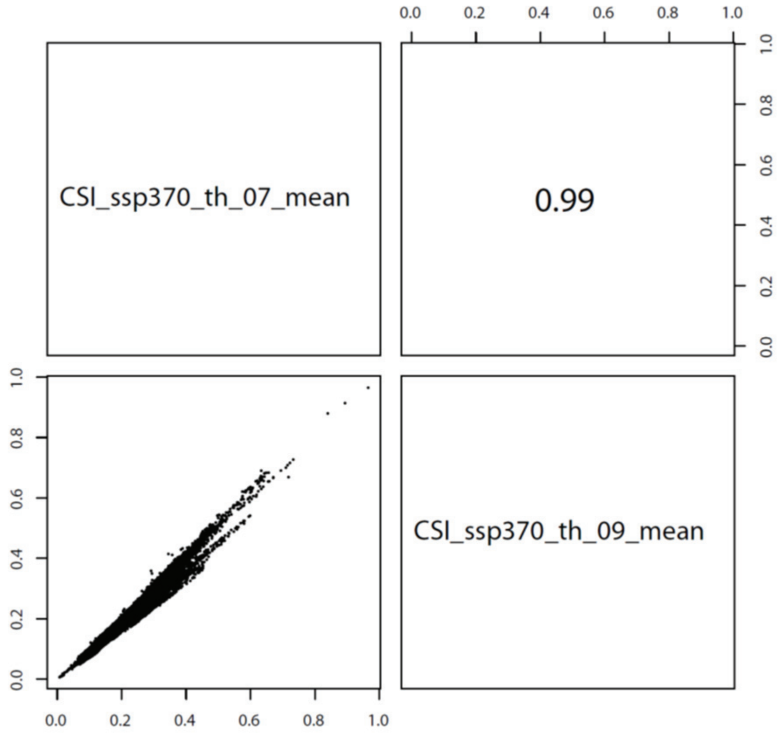

threshold $r = 0.8$ vs $r = 0.9$

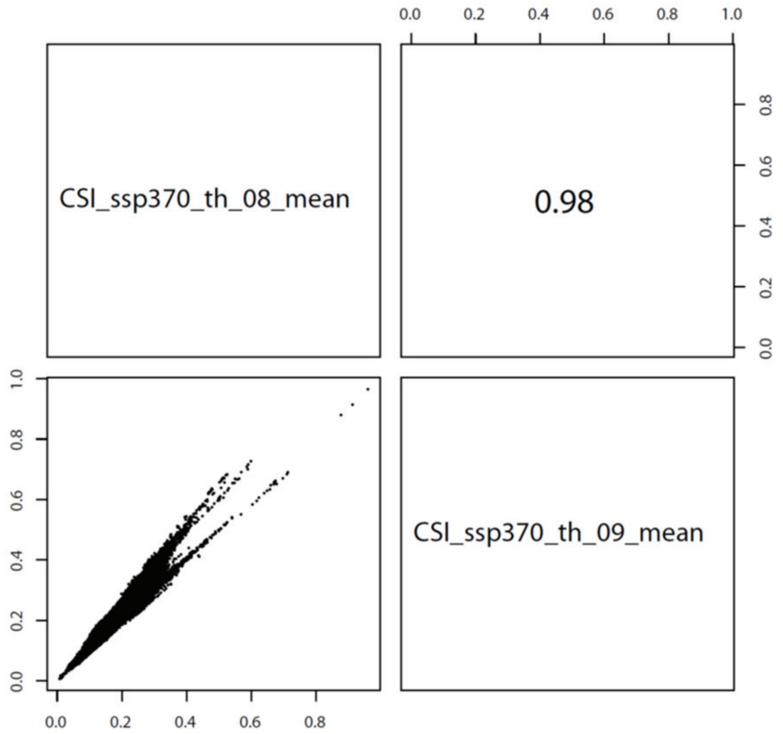

threshold $r = 0.7$ vs $r = 0.8$

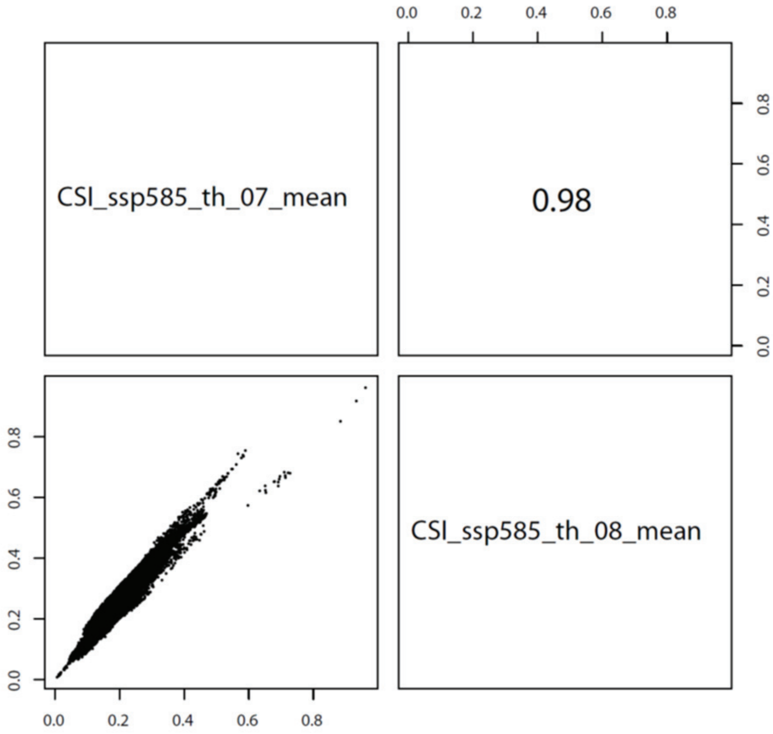

threshold $r = 0.7$ vs $r = 0.9$

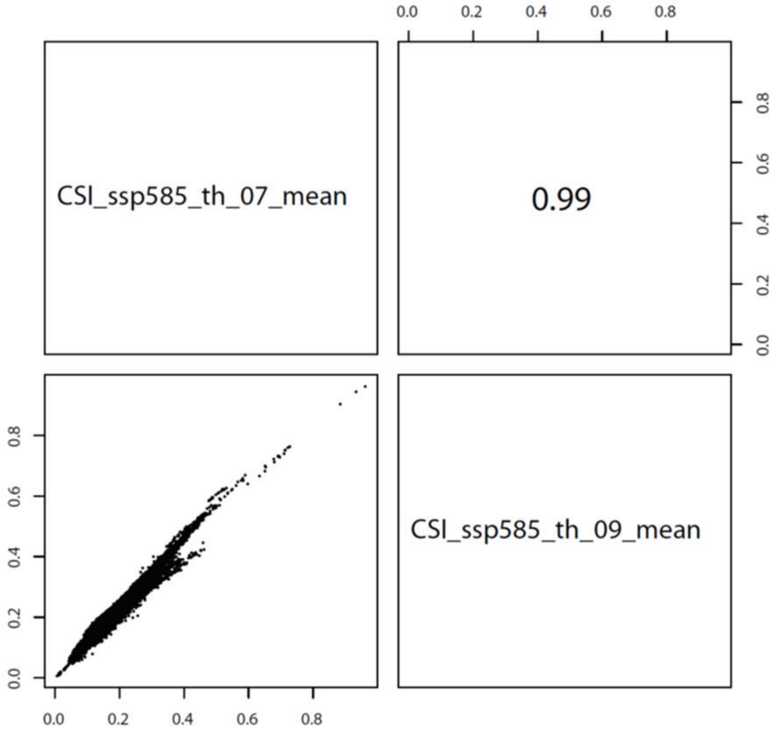

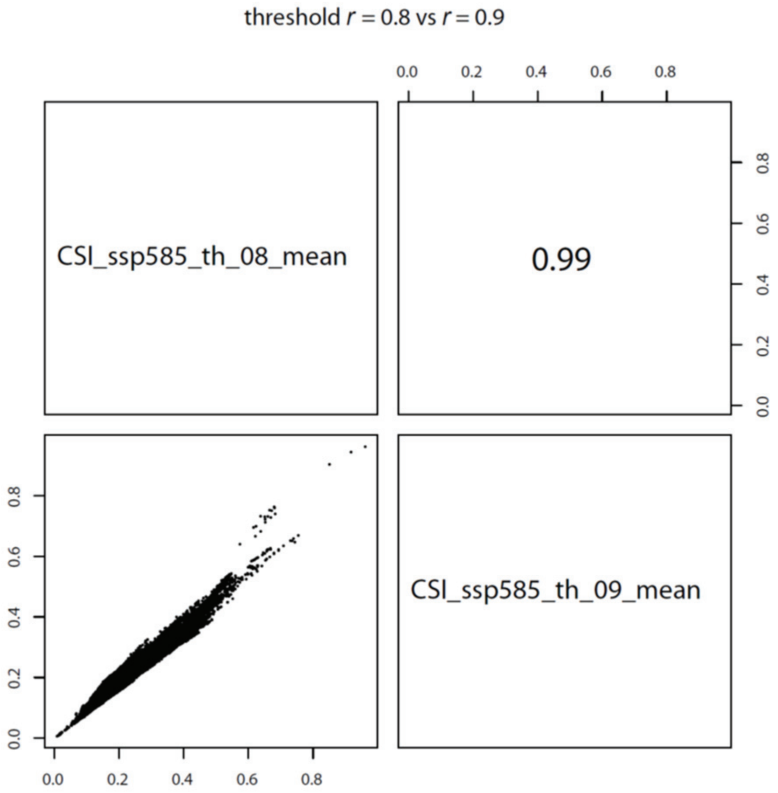
threshold r = 0.8 vs r = 0.9
CSI_ssp585_th_08_mean
0.99
CSI_ssp585_th_09_mean
0.0
0.2
0.4
0.6
0.8

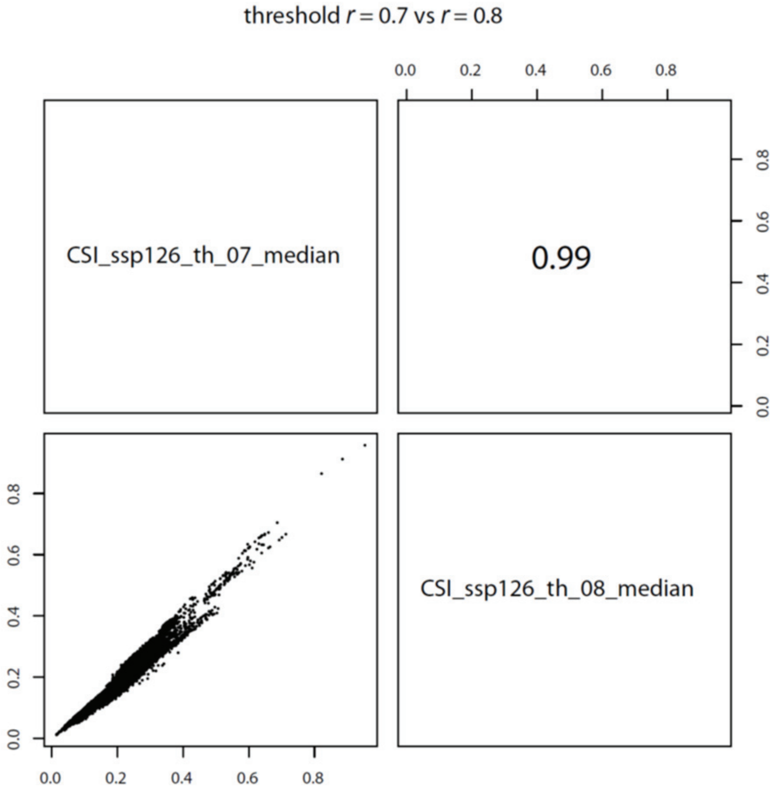
threshold r = 0.7 vs r = 0.8
CSI_ssp126_th_07_median
0.99
CSI_ssp126_th_08_median
0.0
0.2
0.4
0.6
0.8

threshold $r = 0.7$ vs $r = 0.9$

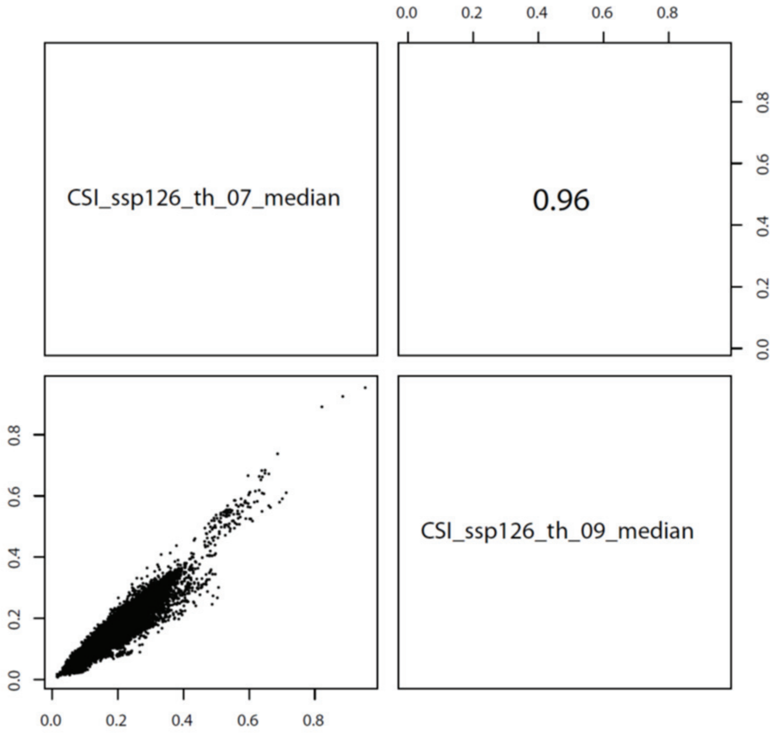

threshold $r = 0.8$ vs $r = 0.9$

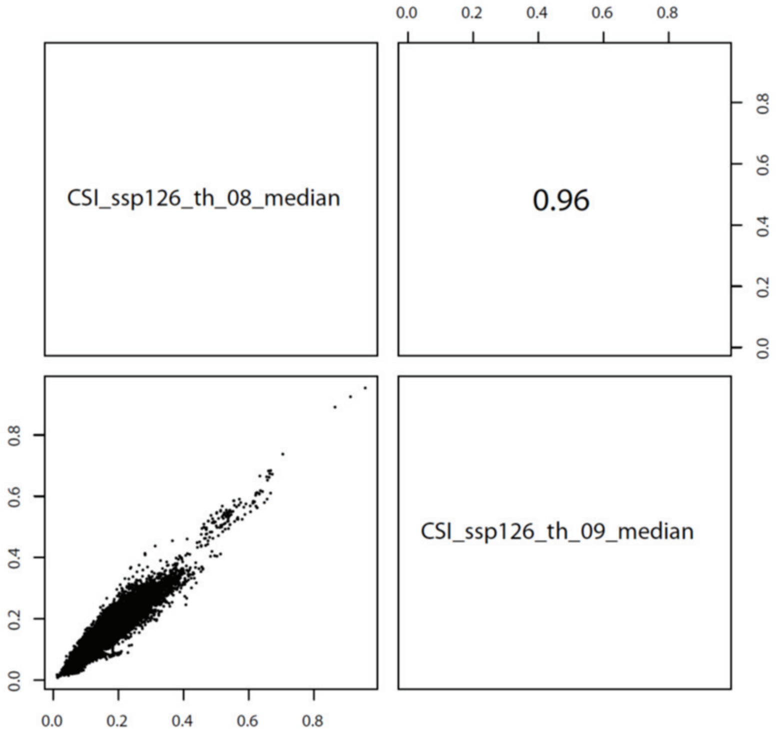

threshold $r = 0.7$ vs $r = 0.8$

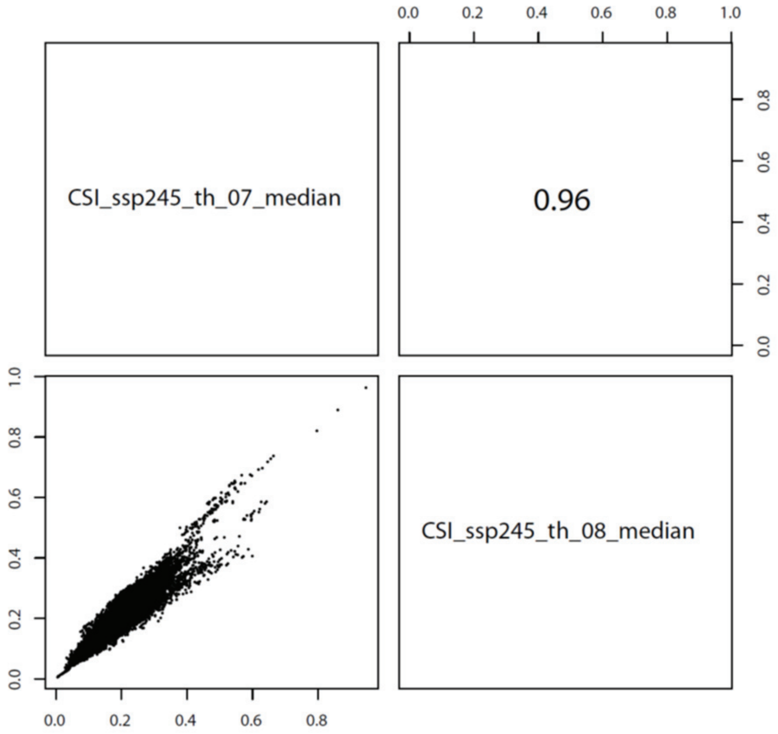

threshold $r = 0.7$ vs $r = 0.9$

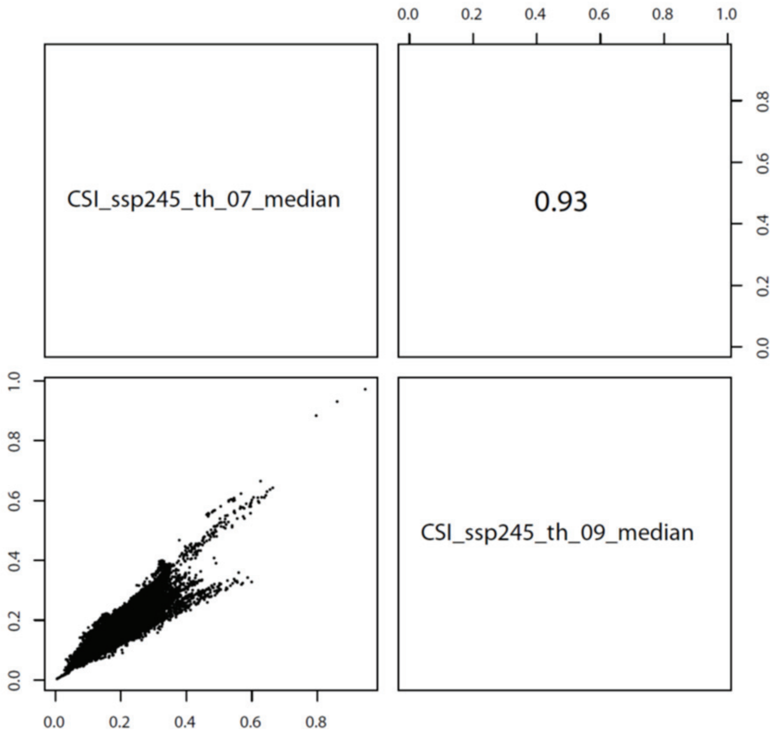

threshold $r = 0.8$ vs $r = 0.9$

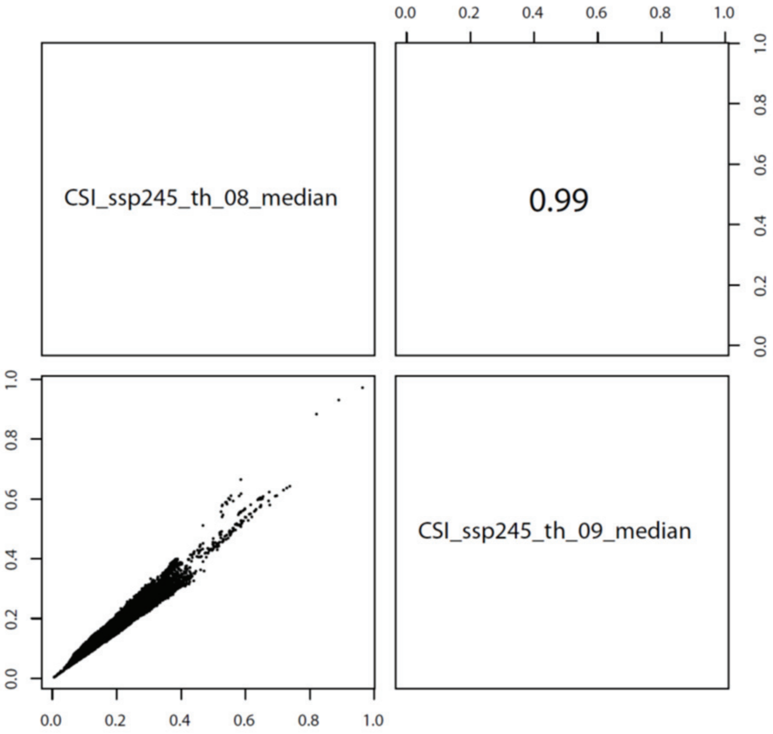

threshold $r = 0.7$ vs $r = 0.8$

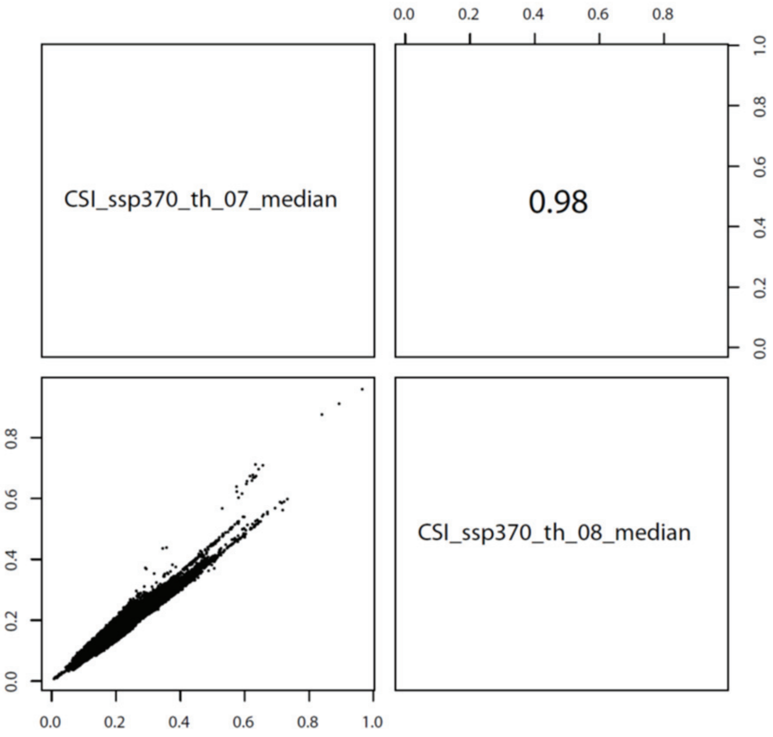

threshold $r = 0.7$ vs $r = 0.9$

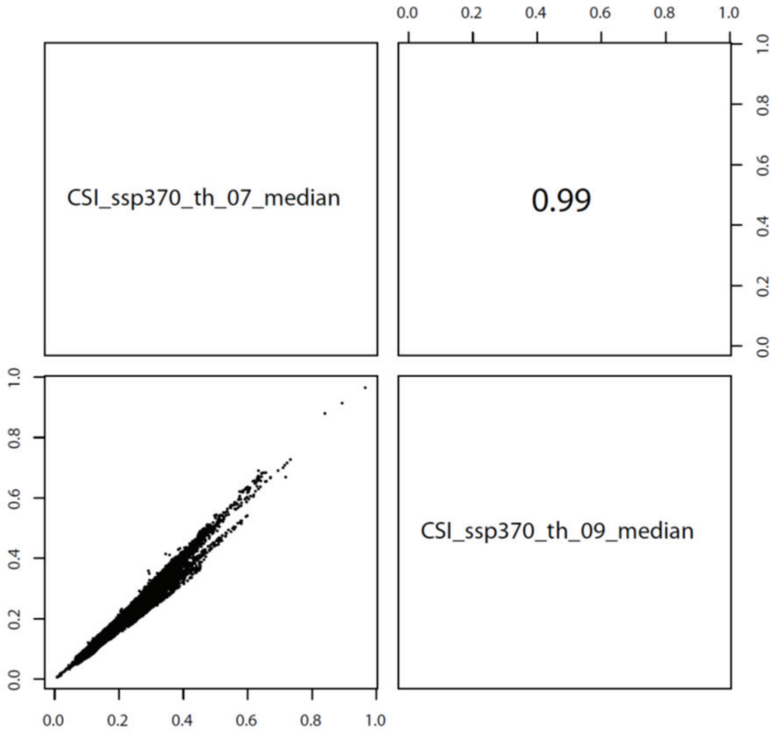

threshold $r = 0.8$ vs $r = 0.9$

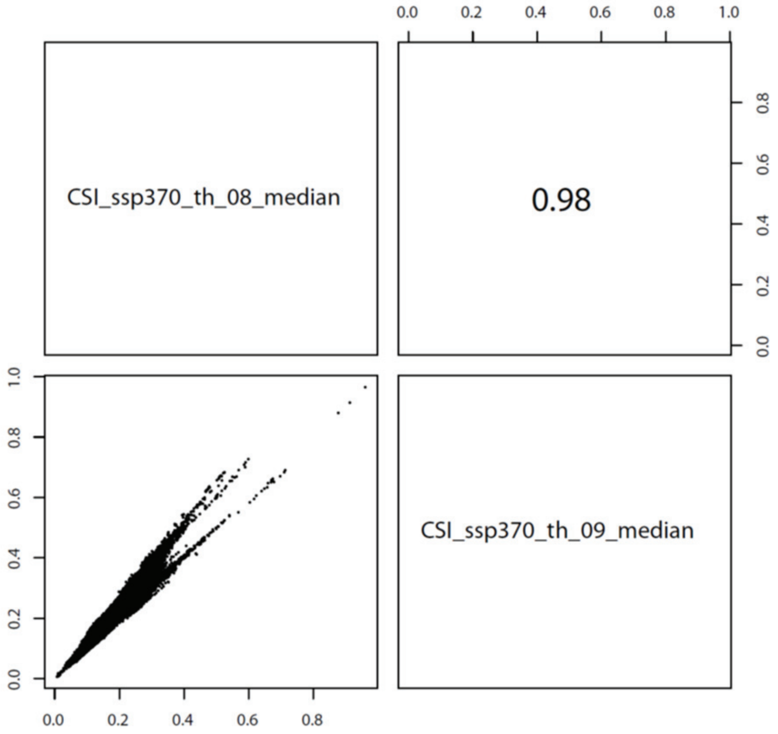

threshold $r = 0.7$ vs $r = 0.8$

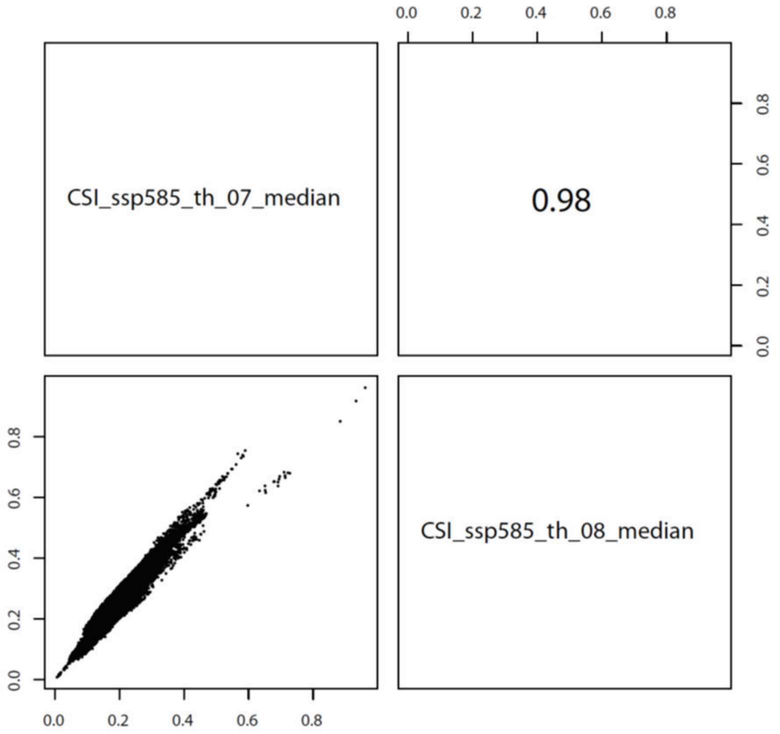

threshold $r = 0.7$ vs $r = 0.9$

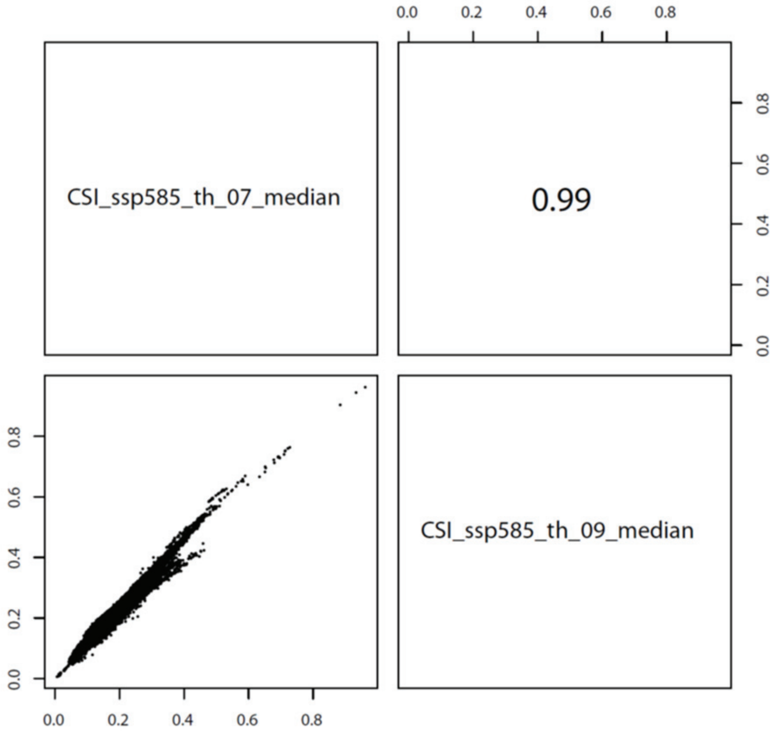

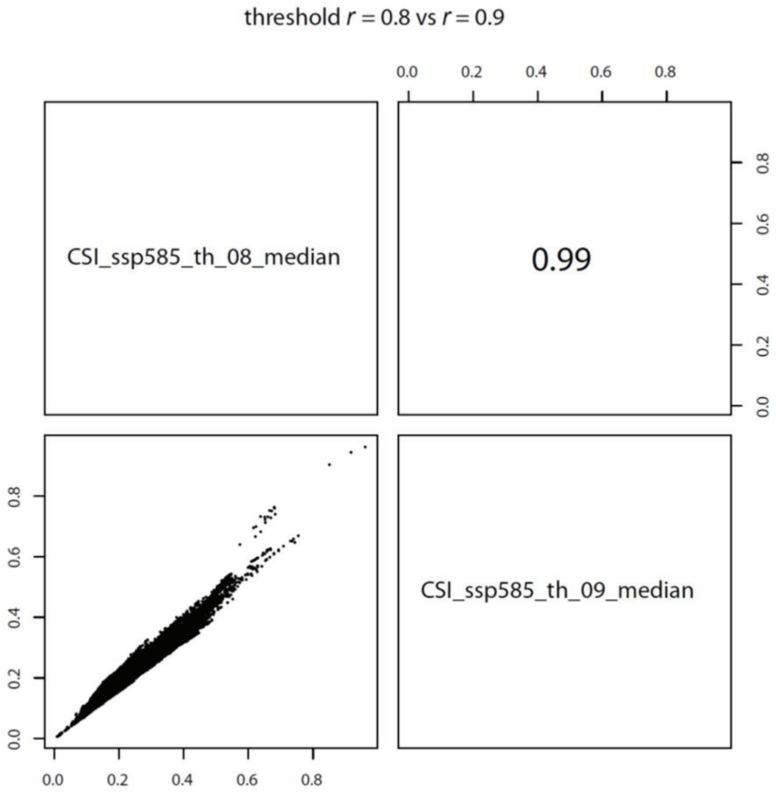
threshold r = 0.8 vs r = 0.9
CSI_ssp585_th_08_median
0.99
CSI_ssp585_th_09_median
0.0
0.2
0.4
0.6
0.8
